# Optimal Intervals of Ultrasonography Screening for Early Diagnosis of Hepatocellular Carcinoma in Taiwan

**DOI:** 10.1001/jamanetworkopen.2021.14680

**Published:** 2021-06-24

**Authors:** Shih-Chiang Kuo, Chia-Ni Lin, Yih-Jyh Lin, Wei-Ying Chen, Jing-Shiang Hwang, Jung-Der Wang

**Affiliations:** 1 Department of Surgery, National Cheng Kung University Hospital, College of Medicine, National Cheng Kung University, Tainan, Taiwan; 2 Department of Public Health, National Cheng Kung University Hospital, College of Medicine, National Cheng Kung University, Tainan, Taiwan; 3 Institute of Clinical Medicine, National Cheng Kung University Hospital, College of Medicine, National Cheng Kung University, Tainan, Taiwan; 4 Department of Internal Medicine, National Cheng Kung University Hospital, College of Medicine, National Cheng Kung University, Tainan, Taiwan; 5 Institute of Statistical Science, Academia Sinica, Taipei, Taiwan; 6 Department of Occupational and Environmental Medicine, National Cheng Kung University Hospital, College of Medicine, National Cheng Kung University, Tainan, Taiwan

## Abstract

**Importance:**

There are different clinical practices regarding ultrasonography screening intervals for hepatocellular carcinoma (HCC) despite recommendations from international guidelines.

**Objective:**

To evaluate whether ultrasonography screening using intervals suggested by international guidelines is associated with cancer stage shifting, reductions in mortality, and improved quality of life (QoL) for patients with HCC.

**Design, Setting, and Participants:**

This nationwide comparative effectiveness research study estimated lifetime survival functions using interlinkages of 3 databases from Taiwan—the Taiwan National Health Insurance, Taiwan National Cancer Registry, and National Mortality Registry—combined with QoL measurements obtained from National Cheng Kung University Hospital. In total, 114 022 patients listed as having newly diagnosed HCC from 2002 through 2015 in the Taiwan National Cancer Registry were followed up until 2017. The QoL values of 1059 patients with HCC who visited National Cheng Kung University Hospital were prospectively measured with the European QoL-5 dimensions questionnaire from 2011 through 2019. Patients were categorized based on the time between their last ultrasonography screening and the index date (90 days prior to HCC diagnosis) as 1 of 5 subcohorts: 6 months (0-6 months), 12 months (7-12 months), 24 months (13-24 months), 36 months (25-36 months), and longer than 36 months (no screening in the previous 3 years). Data were analyzed from April 2020 to April 2021.

**Main Outcomes and Measures:**

Life expectancy, quality-adjusted life expectancy, and loss of life expectancy or loss of quality-adjusted life expectancy compared with age-, sex-, and calendar year–matched cohorts.

**Results:**

There were 59 194 patients with Barcelona Clinic Liver Cancer staging information, including 42 081 men (mean [SD] age, 62.2 [12.6] years) and 17 113 women (mean [SD] age, 69.0 [11.2] years). There was a consistent trend showing that the longer the interval between ultrasonography examinations, the higher the loss of life expectancy and loss of quality-adjusted life expectancy for both sexes. Loss of quality-adjusted life expectancy values for male subcohorts were 10.0 (95% CI, 9.1-10.9) quality-adjusted life-years (QALYs) for ultrasonography screening intervals of 6 months, 11.1 (95% CI, 10.4-11.8) QALYs for 12 months, 12.1 (95% CI, 11.5-12.7) QALYs for 24 months, 13.1 (95% CI, 12.6-13.6) QALYs for 36 months, and 14.6 (95% CI, 14.2-15.0) QALYs for longer than 36 months. Loss of quality-adjusted life expectancy values for female subcohorts were 9.0 (95% CI, 8.3-9.6) QALYs for 6 months, 9.7 (95% CI, 9.2-10.2) QALYs for 12 months, 10.3 (95% CI, 9.8-10.7) QALYs for 24 months, 10.7 (95% CI, 10.2-11.1) QALYs for 36 months, and 11.4 (95% CI, 11.0-11.8) QALYs for longer than 36 months. Patients with underlying hepatitis B virus infection or cirrhosis had the greatest improvement in life expectancy with shorter screening intervals.

**Conclusions and Relevance:**

Regular ultrasonography screening with intervals less than 6 to 12 months may be associated with early detection of HCC, save lives, and improve the quality of life for patients with HCC from a lifetime perspective.

## Key Points

**Question:**

What is the optimal interval of ultrasonography screening for early diagnosis of hepatocellular carcinoma (HCC) among high-risk patients?

**Findings:**

This comparative effectiveness research study evaluated 59 194 patients with HCC in Taiwan who were followed up for 14 years, with loss of quality-adjusted life expectancy estimated by comparison with age-, sex-, and calendar year–matched referents simulated from vital statistics data. Compared with no abdominal ultrasonography screening within 36 months before diagnosis, screening 6 months before diagnosis was associated with an additional 4.6 QALYs for men and 2.4 QALYs for women.

**Meaning:**

In this study, ultrasonography screening using shorter intervals for high-risk patients detected HCC earlier, suggesting that such a screening approach may save more lives and improve quality of life.

## Teaser

This national comparative effectiveness research study estimates differences in life expectancy and quality-adjusted life expectancy for abdominal ultrasonography screening intervals from fewer than 6 months to longer than 3 years among patients at high risk of hepatocellular carcinoma in Taiwan.

## Introduction

Liver cancer is the sixth most common cancer in the world and ranks third in cancer-related mortality, with hepatocellular carcinoma (HCC) accounting for most cases.^1^ Despite improvements in medical technology and treatment in recent years, the 5-year survival rate of HCC remains low.^2,3^ Because survival is associated with cancer stage at diagnosis, early detection by screening has been recommended by international societies, including the American Association for the Study of Liver Diseases, the Asian Pacific Association for the Study of the Liver, and the European Association for the Study of the Liver. They recommend conducting screening programs using abdominal ultrasonography with or without α-fetoprotein at 6-month intervals for high-risk patients.^4,5,6^ Although previous studies have shown that increased frequency of ultrasonography screening for high-risk patients may lead to early detection and improve patient survival,^7,8,9^ several studies do not appear to support this screening recommendation.^10,11,12,13^ Moreover, adherence to regular screenings by high-risk patients has been inadequate,^14,15,16^ leading to reduced overall benefits of ultrasonography screening in real-world practice. In addition, when evaluating screening programs, researchers rarely quantify the quality of life (QoL) and lifetime survival benefits of patients with HCC.

In this study, we explored the comparative effectiveness of different intervals between ultrasonography screenings for detecting HCC at early stages and the association of stage at detection with survival and QoL. Because an expression of the number of life-years or of quality-adjusted life-years (QALYs) associated with detection at early stages may be easily understood by the general public, reporting results by using these measures may increase incentive for regular screening. Thus, we estimated life expectancy (LE), loss of LE, quality-adjusted life expectancy (QALE), and loss of QALE among patients with HCC by using interlinkages with real-world databases.

## Methods

We interlinked the claims data in 3 nationwide databases: Taiwan National Health Insurance, Taiwan Cancer Registry (TCR), and Taiwan Mortality Registry. Personal identification codes were encrypted after linkage, but we obtained patient demographic information, date of diagnosis, cancer site, histopathologic results, survival status, and all outpatient and inpatient health care expenditures covered by Taiwan National Health Insurance. We also used the national life tables of each calendar year from Taiwan’s Vital Statistics for estimation of effectiveness using a lifetime horizon. This study followed the Strengthening the Reporting of Observational Studies in Epidemiology (STROBE) reporting guidelines.^17^ The institutional review board of National Cheng Kung University Hospital approved this study, and we obtained informed consent from every patient who was interviewed for quality of life measurements. No one received compensation or was offered any incentive for participating in this study.

### Establishment of the Cohort of Patients With HCC

The TCR uses *International Classification of Diseases for Oncology, Third Edition* (*ICD-O-3*) codes for coding the site (topography) and the histologic findings (morphology) of neoplasms; the topography codes are essentially identical to those in the *International Classification of Diseases, Tenth Revision, Clinical Modification* (*ICD-10-CM*) system.^18^

It is difficult to determine from claims data whether a patient underwent regular screening before receiving a diagnosis of HCC, especially at an individual level. Therefore, we categorized patients with HCC into different screening subcohorts based on the timing of their last ultrasonography screening before diagnosis, using a method proposed by Wu et al.^8^ We defined the index date for identifying ultrasonography screening for HCC as 90 days prior to the date of diagnosis because ultrasonographic imaging within 3 months before an HCC diagnosis may have been carried out to verify the diagnosis or to serve as an evaluation tool for treatment. We classified patients with HCC into 5 different sex-stratified screening subcohorts based on the timing of the last ultrasonography screening before the index date as follows: 6-month subcohort (0-6 months), 12-month subcohort (7-12 months), 24-month subcohort (13-24 months), 36-month subcohort (25-36 months), and subcohort with longer than 36 months (no ultrasonography screening within 3 years prior to the index date). We also calculated the number of ultrasonography screenings performed within 3 years prior to the index date for each screening subcohort. We estimated the number of previous ultrasonography screenings for individuals who received a diagnosis after April 1, 2003, and categorized them into the aforementioned screening subcohorts to allow for 3 years of accumulation and for 3 months between the diagnosis and the index date because reporting of the claims data used in this study began on January 1, 2000.

We analyzed the underlying liver disease in patients with HCC for each screening subcohort, including hepatitis B virus infection (*ICD-9-CM* code: 070.20-070.23, 070.30-070.33, and V02.61), hepatitis C virus infection (*ICD-9-CM* code: 070.41, 070.44, 070.51, 070.54, 070.70, 070.71, and V02.62), liver cirrhosis (*ICD-9-CM* code: 571.2, 571.5, and 571.6), alcoholic liver disease (*ICD-9-CM* code: 571.0-571.3), and nonalcoholic fatty liver disease (*ICD-9-CM* code: 571.8 and 571.9). We also analyzed major comorbidities that typically result in premature mortality, including ischemic heart disease (*ICD-9-CM* code: 410-414), heart failure (*ICD-9-CM* code: 428), diabetes (*ICD-9-CM* code: 249, 250, 357.2, 362.0, and 366.41), cerebrovascular disease (*ICD-9-CM* code: 430-438), chronic kidney disease (*ICD-9-CM* code: 585), and chronic obstructive pulmonary disease (*ICD-9-CM* code: 491 and 492). For each subcohort, we calculated the proportions of patients who received antiviral therapies for either hepatitis B virus infection or hepatitis C virus infection and for patients with different treatments for HCC after diagnosis, including liver transplantation, liver resection, radiofrequency ablation, percutaneous ethanol injection, transarterial embolization or transarterial chemoembolization, and chemotherapy.

### Cancer Stage Distribution

The TCR typically has 2 staging systems recorded for each patient: the American Joint Committee on Cancer (AJCC) system and the Barcelona Clinic Liver Cancer (BCLC) system. Because BCLC staging was not a required field in the TCR database until 2010, many patients with HCC diagnosed before 2010 had data only for AJCC staging. Because the BCLC staging system incorporates liver function and physical factors into tumor profiles with better predictive power for HCC survival,^19,20^ we analyzed the stage distribution and lifetime survival function based on BCLC staging. We also performed a similar analysis for patients with HCC based on the seventh edition of the AJCC staging system to test robustness and sensitivity.

### Statistical Analysis

#### Estimation of the Lifetime Survival Function, LE, and Loss of LE After Diagnosis

By linking with the Taiwan National Mortality Registry from April 1, 2003, to December 31, 2017, we ascertained the survival status of all patients included in this study for a total of 14.75 years of follow-up. We used the Kaplan-Meier method to estimate the survival function for each HCC stage stratified by sex until the end of follow-up. To extrapolate the survival functions beyond the end of follow-up, we used a rolling extrapolation algorithm with referents simulated using life tables from Taiwan’s National Vital Statistics matched for sex, age, and calendar year of diagnosis.^21^ The rolling extrapolation algorithm is described in eMethods 1 and 2 in the Supplement, with graphic depictions in eFigures 2, 3, 4, and 5 in the Supplement. By integrating the lifetime survival functions, we obtained LE estimates for patients with each HCC stage. Loss of LE was estimated by calculating the difference between the LEs of patients with HCC and matched referents. We applied a bootstrap method to obtain the standard error and 95% CIs of the estimate of loss of LE. All those computations were carried out using the R package iSQoL2. The selected values for input parameters are shown in eTable 1 in the Supplement.^22^ For each ultrasonography screening subcohort, we multiplied the loss of LE value by the proportion of patients in each HCC stage. We then summed all the multiplied values to acquire the weighted mean for the loss of LE for each subcohort.

#### Estimation of QALE and Loss of QALE After Diagnosis

We prospectively and repeatedly collected QoL measurements from 2011 through 2019 for patients with HCC who visited the outpatient departments of the National Cheng Kung University Hospital (NCKUH) by using the European Quality of Life Five-Dimensions (EQ-5D) 3-level questionnaire. The 5 dimensions included in the questionnaire are mobility, self-care, usual activities, pain or discomfort, and anxiety or depression.^23,24^ We transformed the 5 self-reported measurements into a utility value ranging from 0 to 1 by using the scoring function of Taiwan.^25^

We constructed the mean QoL function for patients in each HCC stage by applying kernel smoothing from the time of diagnosis to the maximum assessment time.^26,27^ Lifetime QoL functions were estimated using the extrapolation method described in previous studies.^28,29^ We obtained the QALE for the index cohort of patients in each HCC stage by summing the products of the lifetime survival function and the corresponding mean QoL function across the lifespan. To estimate the mean QoL function of the matched reference cohort, we used the EQ-5D measurements of a representative sample from the 2013 National Health Interview Survey in Taiwan.^30^ Loss of QALE for patients in each HCC stage was calculated by subtracting the values of QALE in patients with HCC (each HCC stage) from sex-, age-, and calendar year–matched referents. The main procedures in our estimation methods are described in eMethods 1 and 2 and eFigures 6, 7, 8, and 9 in the Supplement. Those estimations were computed using the R package iSQoL2 with the selected parameters given in eTable 2 in the Supplement. Similar to the estimation of loss of LE, we weighted the loss of QALE with the proportion of patients in each stage for every ultrasonography screening subcohort and summed them to obtain the loss of QALE for every subcohort. All data analyses were conducted from April 2020 to April 2021.

## Results

We identified 114 022 patients receiving a new diagnosis of HCC between January 1, 2002, and December 31, 2015, with *ICD-O-3* codes for topography of C22.0 and for morphology between 8170 and 8175. Among them, we excluded 14 025 cases for lack of AJCC staging information, age, or date of diagnosis or for miscoding of the date of diagnosis or for patients who developed other malignant neoplasms before or within 1 year after receiving a diagnosis of HCC. In the remaining 99 997 patients, 99 624 patients received a diagnosis after April 2003. Of them, we included 59 194 patients with HCC who had BCLC staging information (eFigure 1 in the Supplement), composed of 42 081 men (71%; mean [SD] age, 62.2 [12.6] years) and 17 113 women (29%; mean [SD] age, 69.0 [11.2] years), in the final analysis (Table 1). Less than one-half of the patients with HCC underwent ultrasonography within either 6 or 12 months before diagnosis (Table 1). Namely, 13 206 (31.4%) male patients with HCC received such screening within 6 months before diagnosis and 16 552 (39.3%) within 12 months before diagnosis, whereas 7224 (42.2%) female patients with HCC received screening within 6 months before diagnosis and 8886 (51.9%) with HCC received such screening within 12 months before diagnosis.

**Table 1.** Baseline Characteristics, Comorbidities, Treatments, and BCLC Stage Distributions, Stratified by Sex and Ultrasonography Screening Interval

Measure	No. (%) of patients									
	Men					Women				
	Ultrasonography screening interval, mo^a^									
	6	12	24	36	>36	6	12	24	36	>36
Total No. of cases	13 206	3346	3168	2136	20 225	7224	1662	1350	858	6019
Age, mean (SD), y	62.1 (11.4)	63.3 (11.9)	63.2 (12.4)	64.4 (12.8)	61.7 (13.4)	68.2 (9.6)	69.4 (10.5)	69.9 (10.6)	70.4 (11.1)	69.4 (13.2)
No. of ultrasonography screens, median, IQR^b,c^	6 (3-8)	3 (2-5)	2 (1-3)	1 (1-1)	0	6 (4-9)	3 (2-5)	2 (1-3)	1 (1-1)	0
Underlying liver disease										
Hepatitis B virus infection	7147 (54.12)	1660 (49.61)	1485 (46.88)	967 (45.27)	9963 (49.26)	2143 (29.67)	455 (27.38)	371 (27.48)	209 (24.36)	1728 (28.71)
Hepatitis C virus infection with cirrhosis	3643 (27.59)	893 (26.69)	696 (21.97)	451 (21.11)	3031 (14.99)	3813 (52.78)	743 (44.71)	540 (40.00)	299 (34.85)	1476 (24.52)
Hepatitis C virus infection and no cirrhosis	1127 (8.53)	340 (10.16)	318 (10.04)	192 (8.99)	1183 (5.85)	790 (10.94)	224 (13.48)	148 (10.96)	114 (13.29)	523 (8.69)
Liver cirrhosis	9875 (74.78)	2217 (66.26)	2035 (64.24)	1317 (61.66)	12 372 (61.17)	5811 (80.44)	1155 (69.49)	923 (68.37)	544 (63.40)	3403 (56.54)
Alcoholic liver disease	1759 (13.32)	408 (12.19)	413 (13.04)	235 (11.00)	2191 (10.83)	178 (2.46)	31 (1.87)	30 (2.22)	19 (2.21)	97 (1.61)
NAFLD	329 (2.49)	92 (2.75)	83 (2.62)	46 (2.15)	325 (1.61)	209 (2.89)	47 (2.83)	40 (2.96)	24 (2.80)	102 (1.69)
Other comorbidities										
Ischemic heart disease	1198 (9.07)	318 (9.50)	287 (9.06)	221 (10.35)	1395 (6.90)	697 (9.65)	178 (10.71)	137 (10.15)	92 (10.72)	495 (8.22)
Heart failure	339 (2.57)	115 (3.44)	105 (3.31)	77 (3.60)	628 (3.11)	296 (4.10)	78 (4.69)	62 (4.59)	50 (5.83)	301 (5.00)
Diabetes	4458 (33.76)	1079 (32.25)	1027 (32.42)	669 (31.32)	4869 (24.07)	2586 (35.80)	590 (35.50)	478 (35.41)	323 (37.65)	1907 (31.68)
Cerebrovascular disease	870 (6.59)	231 (6.90)	243 (7.67)	165 (7.72)	1245 (6.16)	458 (6.34)	130 (7.82)	97 (7.19)	69 (8.04)	422 (7.01)
Chronic kidney disease	1021 (7.73)	320 (9.56)	221 (6.98)	199 (9.32)	1009 (4.99)	527 (7.30)	133 (8.00)	105 (7.78)	82 (9.56)	363 (6.03)
COPD	594 (4.50)	172 (5.14)	166 (5.24)	110 (5.15)	684 (3.38)	201 (2.78)	49 (2.95)	54 (4.00)	17 (1.98)	162 (2.69)
Antiviral therapy										
Hepatitis B virus infection	4920 (37.26)	954 (28.51)	771 (24.34)	467 (21.86)	5060 (25.02)	1277 (17.68)	215 (12.94)	167 (12.37)	84 (9.79)	694 (11.53)
Hepatitis C virus infection	1723 (13.05)	372 (11.12)	237 (7.48)	111 (5.20)	506 (2.50)	1487 (20.58)	244 (14.68)	111 (8.22)	64 (7.46)	171 (2.84)
Treatment of HCC										
Transplantation	479 (3.63)	77 (2.30)	54 (1.70)	29 (1.36)	249 (1.23)	174 (2.41)	15 (0.90)	20 (1.48)	5 (0.58)	42 (0.70)
Resection	4255 (32.22)	1164 (34.79)	1001 (31.60)	584 (27.34)	4556 (22.53)	1658 (22.95)	392 (23.59)	324 (24.00)	207 (24.13)	1183 (19.65)
RFA	5003 (37.88)	947 (28.30)	689 (21.75)	405 (18.96)	2324 (11.49)	3097 (42.87)	561 (33.75)	346 (25.63)	172 (20.05)	810 (13.46)
PEI	1452 (11.00)	265 (7.92)	228 (7.20)	112 (5.24)	788 (3.90)	961 (13.30)	155 (9.33)	117 (8.67)	53 (6.18)	277 (4.60)
TA(C)E	6464 (48.95)	1538 (45.97)	1512 (47.73)	1001 (46.86)	8442 (41.74)	3589 (49.68)	796 (47.89)	601 (44.52)	357 (41.61)	2306 (38.31)
Chemotherapy	3313 (25.09)	875 (26.15)	847 (26.74)	638 (29.87)	6371 (31.50)	1525 (21.11)	363 (21.84)	295 (21.85)	191 (22.26)	1384 (22.99)
BCLC stage distribution										
0	1479 (11.20)	226 (6.75)	103 (3.25)	60 (2.81)	318 (1.57)	948 (13.12)	139 (8.36)	66 (4.89)	37 (4.31)	126 (2.09)
A	6865 (51.98)	1433 (42.83)	1142 (36.05)	560 (26.22)	3100 (15.33)	3997 (55.33)	766 (46.09)	507 (37.56)	268 (31.24)	1187 (19.72)
B	2237 (16.94)	784 (23.43)	829 (26.17)	625 (29.26)	5540 (27.39)	1035 (14.33)	300 (18.05)	332 (24.59)	207 (24.13)	1546 (25.69)
C	1889 (14.30)	663 (19.81)	779 (24.59)	656 (30.71)	8442 (41.74)	860 (11.90)	325 (19.55)	303 (22.44)	237 (27.62)	2162 (35.92)
D	736 (5.57)	240 (7.17)	315 (9.94)	235 (11.00)	2825 (13.97)	384 (5.32)	132 (7.94)	142 (10.52)	109 (12.70)	998 (16.58)

Abbreviations: BCLC, Barcelona Clinic Liver Cancer; COPD, chronic obstructive pulmonary disease; HCC, hepatocellular carcinoma; NAFLD, nonalcoholic fatty liver disease; PEI, percutaneous ethanol injection; RFA, radiofrequency ablation; TA(C)E, transarterial (chemo) embolization.

^a^ Subcohorts categorized based on the timing of the last ultrasonography before the index date (90 days prior to HCC diagnosis): 6, 0 to 6 months; 12, 6 to 12 months; 24, 12 to 24 months; 36, 24-36 months; >36, no screening within 3 years prior to the index date.

^b^ Abdominal ultrasonography screening numbers within 3 years prior to the index date.

^c^ Significant decreasing trend (by Jonckheere-Terpstra trend test) across the 5 screening subcohorts for both sexes (for women, *z* = −128.77; for men, *z* = −209.92; *P* < .001 for both sexes).

For both sexes, there was a significant decreasing trend in the total number of ultrasonographic screens in the 3 years before HCC diagnosis (for women, *z* = −128.77; for men, *z* = −209.92; *P* < .001 for both sexes). Male patients showed higher proportions of underlying hepatitis B virus infection and alcoholic liver disease than female patients, whereas female patients had higher proportions of hepatitis C virus infection and liver cirrhosis. Among patients who did not receive ultrasonography screening within 3 years before diagnosis, more than half had underlying liver cirrhosis (12 372 men [61.2%] and 3403 for women [56.5%]). Patients in subcohorts with shorter screening intervals between HCC diagnosis and the most recent ultrasonography screen typically had higher chances of receiving surgical treatment and locoregional therapies (radiofrequency ablation or percutaneous ethanol injection) for HCC compared with patients in the subcohort having a screening interval longer than 36 months.

For both sexes, the proportions of patients with HCC classified as being in earlier stages (stage 0 and A) were higher in subcohorts with shorter screening intervals since the most recent ultrasonography. In other words, cancer stage shifting occurred across the 5 groups of subcohorts. We also analyzed the stage distribution among patients with HCC after stratification by underlying liver diseases, and the results are summarized in eTable 3 in the Supplement.

### Characteristics and Utility Values for Patients With HCC and QoL Measurements

From 2011 to 2019, a total of 807 men and 252 women diagnosed as having HCC with BCLC staging information received 3370 repeated assessments of QoL for men and 1044 repeated assessments of QoL for women (Table 2). In general, patients with HCC included in the QoL measurements were younger than those abstracted from the national TCR (Table 3). Male patients with QoL measurements were approximately 4 to 13 years younger than Taiwan’s national cohort, whereas female patients were 5 to 9 years younger. The mean QoL utility values during the first, second, and third year or longer after HCC diagnosis are given in Table 2. In general, male patients showed higher QoL utility values than female patients with the same cancer stage. The QoL utility values appeared to be lowest during the first year after diagnosis but improved slightly over time, with the exception of female patients with BCLC stage C, who showed a consistently decreasing trend in QoL utility values. Male patients with more advanced stages appeared to show lower utility values. No women with BCLC stage D were available at NCKUH for QoL measurements.

**Table 2.** Quality of Life Utility Measured in Patients With Hepatocellular Carcinoma Visiting NCKUH From 2011 to 2019, Stratified by Sex and BCLC Stage

Stage	Sex	No. of cases	Age at diagnosis, mean (SD), y	Measurement^a^					
				1st year		2nd year		≥3rd year	
				No.	Utility, mean (SD)	No.	Utility, mean (SD)	No.	Utility, mean (SD)^b^
0	Male	106	56.3 (8.8)	96	0.94 (0.11)	96	0.96 (0.07)	227	0.95 (0.12)
	Female	42	62.2 (9.5)	45	0.87 (0.16)	34	0.95 (0.09)	90	0.92 (0.13)
A	Male	298	58.6 (10.2)	286	0.91 (0.14)	271	0.95 (0.09)	825	0.94 (0.11)
	Female	116	63.6 (11.1)	98	0.83 (0.18)	101	0.88 (0.16)	327	0.91 (0.14)
B	Male	252	59.6 (10.9)	269	0.91 (0.13)	264	0.93 (0.15)	568	0.93 (0.14)
	Female	71	64.7 (11.2)	78	0.87 (0.15)	57	0.90 (0.14)	149	0.89 (0.16)
C	Male	138	56.7 (10.9)	189	0.80 (0.24)	80	0.89 (0.14)	109	0.89 (0.17)
	Female	23	60.8 (9.9)	34	0.86 (0.21)	15	0.84 (0.14)	16	0.79 (0.23)
D	Male	13	50.8 (9.3)	26	0.88 (0.20)	19	0.94 (0.10)	45	0.96 (0.07)
	Female	0^c^	NA	NA	NA	NA	NA	NA	NA

Abbreviations: BCLC, Barcelona Clinic Liver Cancer; EQ-5D, European quality of life-5 dimensions; NA, not applicable; NCKUH, National Cheng Kung University Hospital.

^a^ Utility values were repeatedly measured using EQ-5D questionnaires and converted into a Taiwan value set.^26^

^b^ The mean value of all the utilities measured from the beginning of the third year until the end of follow-up.

^c^ There were no EQ-5D measurements for women with BCLC stage D.

**Table 3.** Loss of LE and Loss of QALE Among Patients With HCC Followed up From 2003 Through 2017, Stratified by Sex and BCLC Stage

BCLC stage	Sex	No. of cases	Age at diagnosis, mean (SD), y	Censored cases, No. (%)	LE (95% CI), y	Loss of LE (95% CI), y	QALE (95% CI), QALYs	Loss of QALE (95% CI), QALYs
0	Male	2186	60.4 (11.3)	1788 (81.8)	15.8 (11.9-19.6)	6.4 (2.6-10.3)	14.8 (11.1-18.5)	6.2 (2.6-9.9)
	Female	1316	67.0 (10.0)	1023 (77.7)	11.8 (9.0-14.7)	8.3 (5.4-11.2)	10.8 (8.2-13.5)	6.8 (4.1-9.5)
A	Male	13 100	62.0 (11.7)	7991 (61.0)	13.5 (12.1-15.0)	7.4 (6.0-8.9)	12.8 (11.4-14.2)	7.0 (5.6-8.5)
	Female	6725	67.9 (10.2)	3779 (56.2)	10.3 (9.4-11.2)	9.2 (8.2-10.1)	9.4 (8.5-10.3)	7.6 (6.7-8.5)
B	Male	10 015	63.6 (12.7)	3605 (36.0)	7.8 (6.8-8.7)	12.2 (11.2-13.1)	7.2 (6.4-8.0)	11.6 (10.8-12.4)
	Female	3420	69.0 (11.9)	1094 (32.0)	5.8 (5.0-6.6)	12.9 (12.0-13.7)	5.1 (4.4-5.9)	11.1 (10.3-11.9)
C	Male	12 429	61.2 (13.0)	1454 (11.7)	2.5 (1.8-3.2)	19.3 (18.6-20.1)	2.2 (1.6-2.8)	18.4 (17.7-19.1)
	Female	3887	69.8 (12.0)	560 (14.4)	3.1 (2.4-3.8)	15.1 (14.3-15.8)	2.5 (1.9-3.2)	13.3 (12.6-14.0)
D	Male	4351	63.3 (13.9)	161 (3.7)	1.1 (0.8-1.5)	19.3 (18.8-19.8)	1.1 (0.7-1.4)	18.2 (17.8-18.7)
	Female	1765	73.0 (11.8)	88 (5.0)	0.8 (0.6-0.9)	14.9 (14.5-15.4)	0.6 (0.5-0.8)^a^	12.8 (12.5-13.2)

Abbreviations: BCLC, Barcelona Clinic Liver Cancer; HCC, hepatocellular carcinoma; LE, life expectancy; QALE, quality-adjusted life expectancy; QALY, quality-adjusted life-years.

^a^ QALE and loss of QALE among female patients with BCLC stage D were calculated using the utility value for female patients with BCLC stage C.

### LE, Loss of LE, QALE, and Loss of QALE, Stratified by HCC Stage and Sex

By assuming the QoL function of female patients with BCLC stage D to be similar to stage C, we estimated QALE and loss of QALE for the former subcohort. Table 3 gives the age distribution, LE, loss of LE, QALE, and loss of QALE values for patients with HCC from the national cohort included in this study, stratified by sex and BCLC stage. For both sexes, we found a consistent decrease in LE and QALE as the cancer stage progressed. There was a trend for increasing loss of LE and loss of QALE from BCLC stage 0 to stage C (Figure). However, loss of LE and loss of QALE were similar for patients with HCC stage C and stage D.

**Figure.**
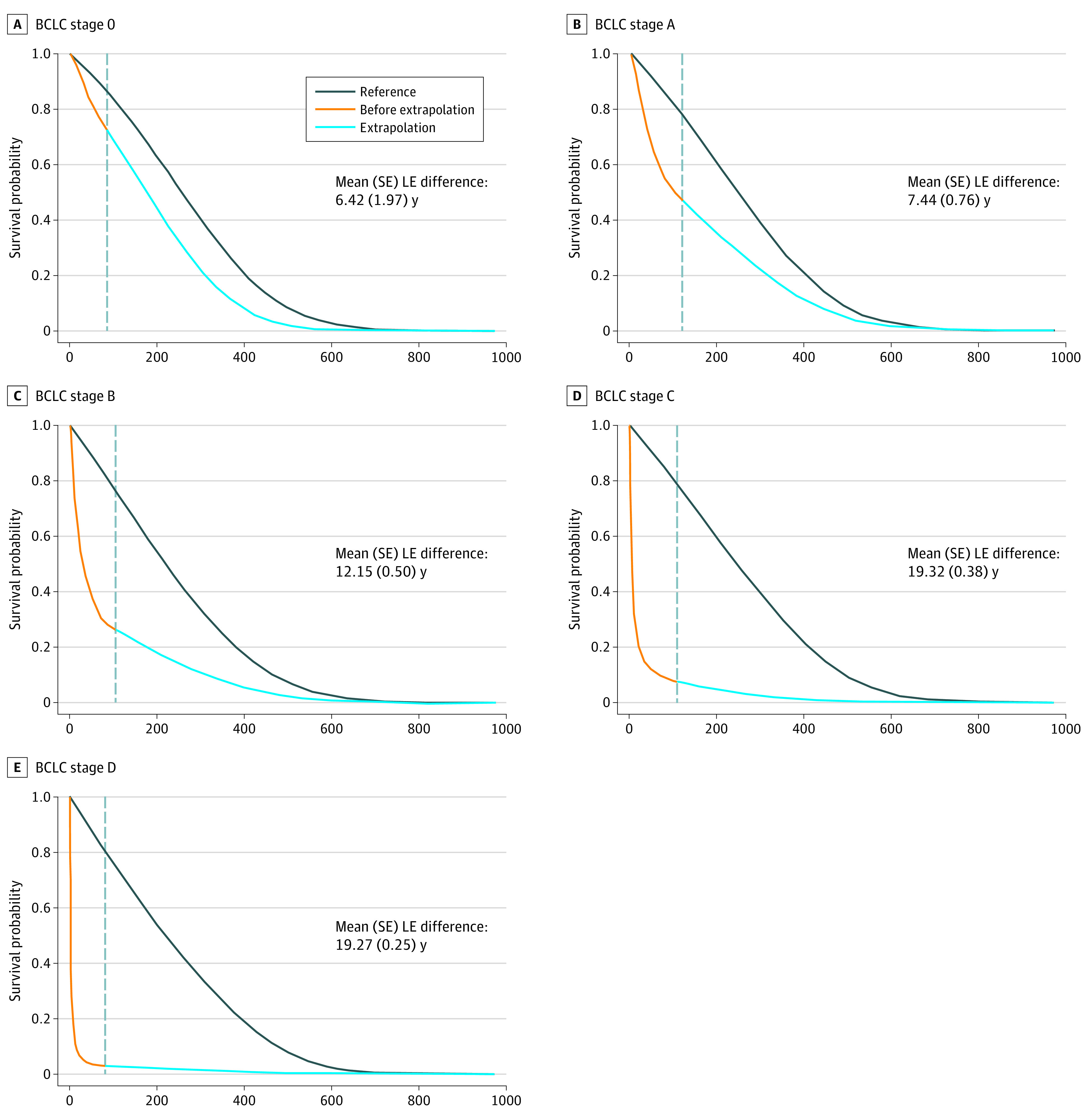
Loss of Life Expectancy for Men With Hepatocellular Carcinoma Stratified by Barcelona Clinic Liver Cancer (BCLC) Stage The gray dashed vertical line in each panel indicates the last month (*F*) before the extrapolation of survival functions. We selected a month close to the end of follow-up, depending on the sparsity of the observed death events. The values of *F* in months for these men are 85 for stage 0, 117 for stage A, 105 for stage B, 109 for stage C, and 82 for stage D (eTable 1 in the Supplement). The area between the 2 curves in each panel represents the mean difference in life expectancies (LEs) between men with hepatocellular carcinoma of a specific stage (area below the orange and blue lines) and that of the sex-, age-, and calendar year–matched referents simulated from life tables obtained using Taiwan’s National Vital Statistics (area below the black line).

### Loss of LE and Loss of QALE for Different Screening Intervals

Table 4 gives the overall loss of LE and loss of QALE for each ultrasonography screening subcohort weighted by stage distribution. There was a consistent trend showing that the longer the interval between the last ultrasonography examination and HCC diagnosis, the higher the loss of LE and loss of QALE for both sexes. Loss of QALE values for men were 10.0 (95% CI, 9.1-10.9) QALYs for the 6-month subcohort, 11.1 (95% CI, 10.4-11.8) QALYs for the 12-month subcohort, 12.1 (95% CI, 11.5-12.7) QALYs for the 24-month subcohort, 13.1 (95% CI, 12.6-13.6) QALYs for the 36-month subcohort, and 14.6 (95% CI, 14.2-15.0) QALYs for the subcohort with longer than 36 months. Loss of QALE (95% CI) values for women were 9.0 (95% CI, 8.3-9.6) QALYs for 6 months, 9.7 (95% CI, 9.2-10.2) QALYs for 12 months, 10.3 (95% CI, 9.8-10.7) QALYs for 24 months, 10.7 (95% CI, 10.2-11.1) QALYs for 36 months, and 11.4 (95% CI, 11.0-11.8) QALYs for longer than 36 months. Compared with patients screened at an interval longer than 36 months, patients screened between 0 and 6 months before the index date was associated with an additional 4.8 life-years or 4.6 QALYs for male patients and 2.6 life-years or 2.4 QALYs for female patients.

**Table 4.** Loss of LE and Loss of QALE for Different HCC Ultrasonography Screening Intervals Weighted by BCLC Stage Distribution and Stratified by Sex

Underlying liver disease	Measurement	Loss (95% CI)				
		Ultrasonography screening interval, mo^a^				
		6	12	24	36	>36
All patients						
Male	Loss of LE	10.5 (9.6-11.4)	11.7 (10.9-12.4)	12.7 (12.1-13.4)	13.7 (13.2-14.3)	15.3 (14.8-15.9)
	Loss of QALE	10.0 (9.1-10.9)	11.1 (10.4-11.8)	12.1 (11.5-12.7)	13.1 (12.6-13.6)	14.6 (14.2-15.0)
Female	Loss of LE	10.6 (9.9-11.3)	11.4 (10.8-11.9)	12.0 (11.5-12.5)	12.4 (11.9-12.8)	13.2 (12.8-13.6)
	Loss of QALE	9.0 (8.3-9.6)	9.7 (9.2-10.2)	10.3 (9.8-10.7)	10.7 (10.2-11.1)	11.4 (11.0-11.8)
Hepatitis B virus infection						
Male		7147 (33.7)	1660 (7.8)	1485 (7.0)	967 (4.6)	9963 (47.0)
	Loss of LE	10.1 (9.2-11.1)	11.3 (10.5-12.1)	12.4 (11.7-13.0)	13.4 (12.8-13.9)	15.2 (14.7-15.6)
	Loss of QALE	9.6 (8.7-10.5)	10.7 (10.0-11.5)	11.8 (11.2-12.4)	12.7 (12.2-13.3)	14.4 (14.0-14.9)
Female		2143 (43.7)	455 (9.3)	371 (7.6)	209 (4.3)	1728 (35.2)
	Loss of LE	10.3 (9.6-11.1)	11.1 (10.5-11.7)	11.7 (11.2-12.2)	12.1 (11.6-12.6)	13.1 (12.7-13.6)
	Loss of QALE	8.7 (8.0-9.4)	9.5 (8.9-10.0)	10.0 (9.5-10.5)	10.4 (9.9-10.8)	11.3 (10.9-11.8)
HCV infection with cirrhosis						
Male		3643 (41.8)	893 (10.2)	696 (8.0)	451 (5.2)	3031 (34.8)
	Loss of LE	10.3 (9.4-11.2)	11.7 (11.0-12.5)	12.4 (11.7-13.1)	13.8 (13.2-14.3)	14.8 (14.4-15.3)
	Loss of QALE	9.8 (8.9-10.7)	11.2 (10.4-11.9)	11.8 (11.1-12.4)	13.1 (12.6-13.6)	14.1 (13.7-14.6)
Female		3813 (55.5)	743 (10.8)	540 (7.9)	299 (4.4)	1476 (21.5)
	Loss of LE	10.6 (9.9-11.3)	11.3 (10.7-11.9)	11.7 (11.2-12.2)	12.4 (11.9-12.8)	12.8 (12.4-13.3)
	Loss of QALE	8.9 (8.3-9.6)	9.6 (9.1-10.2)	10.0 (9.6-10.5)	10.6 (10.2-11.1)	11.1 (10.6-11.5)
HCV infection without cirrhosis						
Male		1127 (35.7)	340 (10.8)	318 (10.1)	192 (6.1)	1183 (37.4)
	Loss of LE	9.3 (8.3-10.4)	10.0 (9.1-10.9)	10.9 (10.1-11.7)	12.3 (11.7-13.0)	13.4 (12.9-14.0)
	Loss of QALE	8.9 (7.9-9.9)	9.5 (8.6-10.4)	10.4 (9.6-11.2)	11.7 (11.1-12.3)	12.8 (12.3-13.3)
Female		790 (43.9)	224 (12.4)	148 (8.2)	114 (6.3)	523 (29.1)
	Loss of LE	10.0 (9.2-10.8)	10.7 (10.0-11.4)	11.2 (10.6-11.8)	11.1 (10.5-11.7)	12.4 (11.9-12.8)
	Loss of QALE	8.4 (7.7-9.2)	9.0 (8.4-9.7)	9.5 (8.9-10.1)	9.4 (8.8-10.0)	10.6 (10.2-11.1)
All patients with cirrhosis						
Male		9875 (35.5)	2217 (8.0)	2035 (7.3)	1317 (4.7)	12 372 (44.5)
	Loss of LE	10.7 (9.8-11.6)	12.1 (11.4-12.8)	13.1 (12.5-13.7)	14.2 (13.7-14.7)	15.7 (15.3-16.2)
	Loss of QALE	10.2 (9.4-11.1)	11.5 (10.9-12.2)	12.5 (11.9-13.0)	13.5 (13.0-14.0)	15.0 (14.5-15.4)
Female		5811 (49.1)	1155 (9.8)	923 (7.8)	544 (4.6)	3403 (28.8)
	Loss of LE	10.7 (10.0-11.3)	11.5 (11.0-12.1)	12.0 (11.6-12.5)	12.6 (12.2-13.0)	13.2 (12.8-13.6)
	Loss of QALE	9.0 (8.4-9.7)	9.8 (9.3-10.3)	10.3 (9.9-10.8)	10.9 (10.4-11.3)	11.4 (11.0-11.8)
No underlying liver disease^b^						
Male		669 (12.4)	288 (5.3)	398 (7.4)	331 (6.1)	3725 (68.8)
	Loss of LE	12.2 (11.5-12.9)	13.4 (12.8-13.9)	14.1 (13.5-14.6)	14.1 (13.6-14.6)	15.5 (15.1-16.0)
	Loss of QALE	11.6 (10.9-12.2)	12.7 (12.1-13.2)	13.4 (12.9-13.9)	13.4 (12.9-13.8)	14.8 (14.4-15.2)
Female		281 (12.4)	161 (7.1)	181 (8.0)	126 (5.6)	1522 (67.0)
	Loss of LE	11.8 (11.2-12.3)	12.0 (11.6-12.5)	12.6 (12.2-13.0)	12.9 (12.5-13.3)	13.5 (13.1-13.9)
	Loss of QALE	10.1 (9.6-10.6)	10.3 (9.9-10.8)	10.8 (10.4-11.2)	11.1 (10.7-11.5)	11.7 (11.3-12.1)

Abbreviations: BCLC, Barcelona Clinic Liver Cancer; HCC, hepatocellular carcinoma; HCV, hepatitis C virus; LE, life expectancy; QALE, quality-adjusted life expectancy.

^a^ Subcohorts are categorized based on the timing of the last ultrasonography before the index date (90 days prior to the HCC diagnosis): 6, 0 to 6 months; 12, 6 to 12 months; 24, 12 to 24 months; 36, 24 to 36 months; >36 months, no screening within 3 years prior to the index date.

^b^ Included patients without any of the following underlying liver diseases: hepatitis B virus infection, hepatitis C virus infection, liver cirrhosis, alcoholic liver disease, and nonalcoholic fatty liver disease.

### Subgroup Analysis

We performed the same analysis after stratification by underlying liver disease, and the results are given in Table 4. The proportions of patients in the 6-month subcohort with liver cirrhosis were 35.5% for men and 49.1% for women. Patients with underlying hepatitis B virus infection or with hepatitis C virus infection combined with liver cirrhosis experienced the most benefits assocaited with shorter intervals of ultrasonography screening. For patients with underlying hepatitis B virus infection, compared with an interval longer than 36 months, the potential savings for the 6-month subcohort was 5.1 life-years or 4.8 QALYs for men and 2.8 life-years or 2.6 QALYs for women. For patients with underlying liver cirrhosis, compared with an interval longer than 36 months, the potential savings for the 6-month subcohort was 5.0 life-years or 4.8 QALYs for men and 2.5 life-years or 2.4 QALYs for women. For patients without any underlying liver disease listed in Table 1, compared with an interval longer than 36 months, the potential savings for the 6-month subcohort was still 3.3 life-years or 3.2 QALYs for men and 1.7 life-years or 1.6 QALYs for women.

### Sensitivity Analysis

We performed the same analysis for 99 624 patients with HCC by using the seventh edition of the AJCC staging system (eTables 4 and 7 in the Supplement). Patients with QoL measurements from NCKUH were also stratified by data using this AJCC staging system, and eTable 5 in the Supplement gives their QoL utility values. The loss of LE and loss of QALE for patients in each stage and the overall ultrasonography screening subcohort are provided in eTables 6 and 8 in the Supplement. We found that the loss of QALE consistently increased with longer screening intervals for the most recent ultrasonography, although the magnitude was slightly smaller using this staging system vs using the BCLC system.

## Discussion

We estimated survival and QoL utility in patients with HCC using a lifetime horizon and found that a shorter ultrasonography screening interval was associated with cancer stage shifting and with savings in life-years or loss of QALE. Our study has the following strengths. First, we included only HCC cases with *ICD-O-3* topography code C22.0 and *ICD-O-3* morphology codes between 8170 and 8175. By doing so, we excluded approximately 1.9% of HCC cases, but all the diagnoses of HCC were valid. Because all patients with HCC are waived from making copayments for health care in the Taiwan National Health Insurance system, the data abstracted from the national registries are comprehensive. Second, we followed up the study cohorts for 14.75 years, which is longer than LEs of patients with HCC except for male patients with BCLC stage 0. Moreover, using a month-to-month rolling extrapolation to estimate the lifetime survival function after the end of follow-up is more accurate than using a parametric estimation.^21^ Third, the stage shifting apparent in Table 1 was consistent with our hypothesis that ultrasonography screening with a shorter interval, for example, every 6 or 12 months, for high-risk patients would lead to early detection of HCC and lower mortality rates.^8^ When we stratified the larger cohort of 99 624 patients with HCC using the seventh edition of the AJCC staging system, the results consistently showed stage shifting for shorter intervals of ultrasonography screening (eTables 4, 5, 6, 7, and 8 in the Supplement). Fourth, stage stratification made the HCC subcohorts in each stage more homogeneous and less confounded by different socioeconomic status or levels of health literacy. Because the proportion of patients who received transplantation for treatment of HCC in the 6-month interval subcohort was approximately only 1% to 2% as high as all the other subcohorts (Table 1), this proportion cannot account for the large health benefits observed in this subcohort (Table 4). All of these findings provide supporting evidence for the validity of this study. Fifth, the estimations and comparisons of loss of LE and loss of QALE as health impacts were adjusted for different distributions of age, sex, and calendar year of medical technology (eFigure 10 in the Supplement), which reduces the potential lead time bias resulting from younger patients for earlier stages of HCC. Moreover, we stratified by cancer stage and estimated loss of LE and loss of QALE at different stages, which adjusts for different speeds of progression of HCC. After being weighted by the proportions of different stages, the sum of loss of LE minimizes length time bias. Loss of LE and loss of QALE values are likely more comprehensible to the general public than the 5-year mortality or median survival used in previous studies,^3,31,32^ which may facilitate patient participation in decision-making. Finally, we combined the utility values of QoL and survival of patients with HCC, enabling us to estimate loss of QALE, or lifetime utility loss, for the screening subcohorts. The health benefits of screening can be compared with other health care technologies for resource allocation. We thus tentatively conclude that a shorter screening interval, as frequent as every 6 months, saves lives and lifetime utility for patients with HCC.

Although international organizations and the Taiwan Liver Cancer Association and the Gastroenterological Society of Taiwan^33^ recommend ultrasonography screening for HCC every 6 to 12 months, our study found that only 31.4% of men and 42.2% of women with HCC in Taiwan underwent ultrasonography within 6 months before diagnosis, and only 39.3% of males and 51.9% of females underwent such screening within 12 months. Moreover, among those with underlying liver cirrhosis, only 35.5% of men (9875 of 27 816) and 49.1% of women (5811 of 11 836) underwent ultrasonography within 6 months before HCC diagnosis, indicating underutilization of ultrasonography screening in Taiwan (Table 4).

That 61.2% of men and 56.5% of women in the subcohort with a screening interval longer than 36 months had underlying liver cirrhosis but none of them underwent ultrasonography within the 3 years prior to their diagnosis of HCC deserves further attention. Compared with male patients, there was a higher proportion of female patients in the 6-month subcohort and a lower proportion in subcohort with longer than 36 months. This sex difference may be attributed to an overall higher level of health literacy among women in Taiwan, but we were unable to clarify the cause in this study.

### Limitations

This study has limitations. First, the patients categorized into the 6- or 12-month screening subcohorts did not necessarily receive regular ultrasonography screening because the categorization only considered the last ultrasonography performed before the index date. Second, patients with HCC having QoL measurements obtained at NCKUH were from outpatient departments and were generally younger than the national HCC population. Therefore, the utility values and QALE in this study would be overestimated, and the loss of QALE would be underestimated. In particular, because there were no QoL measurements for female patients with BCLC stage D, the calculation of their QALE using information from female patients with BCLC stage C would most likely cause an overestimation, leading to a greater underestimation of loss of QALE for female patients with stage D HCC. In addition, our study estimated loss of LE and loss of QALE for patients with HCC, and we did not consider individuals who underwent ultrasonography screening but never developed HCC, which may lead to a slight overestimation of the overall health benefit of the surveillance program.

## Conclusions

The results of this study suggest that regular ultrasonography screening with an interval of 6 to 12 months or less may lead to early detection of HCC and may save lives and improve utility for patients with HCC from a lifetime perspective. Because people with underlying risk factors (including hepatitis B virus or hepatitis C virus infection, cirrhosis, and alcoholic liver disease) showed only slightly more frequent ultrasonography screening than those without underlying risk factors, we recommend improving this clinical practice.

## Notes

Supplement. **eFigure 1.** Flow Diagram of Study Cohort **eMethods 1.** Theoretical Part of the Statistical Methods **eMethods 2.** Empirical Part of the Statistical Methods **eFigure 2.** Kaplan-Meier Survival Estimates for Stage A Male HCC Patients and the Corresponding Referents **eFigure 3.** Logit Transformation of Survival Ratio *W(t)* Between Index Cohort and Referents Fitted with a Restricted Cubic Spline Model **eFigure 4.** Rolling-Over Extrapolation of logit[*W(t)*] to Lifetime **eFigure 5.** Lifetime Survival Function of Stage A Male HCC Patients and the Age-, Sex-, Calendar Year-Matched Referents **eTable 1.** Parameters Selected for Rolling Extrapolation of Survival Functions of HCC Patients **eFigure 6.** Kernel-Smoothing Mean of QoL for Stage A Male HCC Patients, From Diagnosis to the Maximum Time *Fq* **eFigure 7.** Average QoL of Stage A Male HCC Patients in *K* Months Before Death **eFigure 8.** QALE, the Integration of Lifetime Survival Function S ^(t│index) and Lifetime QoL Function Q ^(t│index) **eFigure 9.** Loss-of-QALE of the Stage A Male HCC Patients **eTable 2.** Parameters Selected for QALE Estimation of HCC Patients **eTable 3.** Frequency Distribution of HCC Subcohorts Stratified by Gender, Different Underlying Liver Disease, and BCLC Stage **eTable 4.** Baseline Characteristics, Comorbidities, and AJCC 7th Stage Distributions of Different HCC Subcohorts Stratified by Gender **eTable 5.** QoL Utility Measured in HCC Patients of NCKUH Stratified by Gender and AJCC 7th Staging (2011-2019) **eTable 6.** Loss-of-LE and Loss-of-QALE of HCC Patients Stratified by Gender and AJCC 7th Staging, Followed During 2002-2017 **eTable 7.** Frequency Distribution of HCC Subcohorts Stratified by Gender, Different Underlying Liver Disease, and AJCC 7th Staging **eTable 8.** Loss-of-LE and Loss-of-QALE of Different HCC Subcohorts Weighted by AJCC 7th Stage Distribution **eFigure 10.** Adjustment of Age, Sex, and Calendar-Year of Diagnosis by Estimating the Difference-in-Differences of Loss-of-LE eReferences.
