# Supplementary material for: Optimal Intervals of Ultrasonography Screening for Early Diagnosis of Hepatocellular Carcinoma in Taiwan
Source: JAMA Netw Open. 2021 Jun 24;4(6):e2114680. doi: 10.1001/jamanetworkopen.2021.14680 (PMC8226422; doi:10.1001/jamanetworkopen.2021.14680)
Supplement: Supplement. — eFigure 1. Flow Diagram of Study Cohort eMethods 1. Theoretical Part of the Statistical Methods eMethods 2. Empirical Part of the Statistical Methods eFigure 2. Kaplan-Meier Survival Estimates for Stage A Male HCC Patients and the Corresponding Referents eFigure 3. Logit Transformation of Survival Ratio W(t) Between Index Cohort and Referents Fitted with a Restricted Cubic Spline Model eFigure 4. Rolling-Over Extrapolation of logit[W(t)] to Lifetime eFigure 5. Lifetime Survival Function of Stage A Male HCC Patients and the Age-, Sex-, Calendar Year-Matched Referents eTable 1. Parameters Selected for Rolling Extrapolation of Survival Functions of HCC Patients eFigure 6. Kernel-Smoothing Mean of QoL for Stage A Male HCC Patients, From Diagnosis to the Maximum Time Fq eFigure 7. Average QoL of Stage A Male HCC Patients in K Months Before Death eFigure 8. QALE, the Integration of Lifetime Survival Function S ^(t│index) and Lifetime QoL Function Q ^(t│index) eFigure 9. Loss-of-QALE of the Stage A Male HCC Patients eTable 2. Parameters Selected for QALE Estimation of HCC Patients eTable 3. Frequency Distribution of HCC Subcohorts Stratified by Gender, Different Underlying Liver Disease, and BCLC Stage eTable 4. Baseline Characteristics, Comorbidities, and AJCC 7th Stage Distributions of Different HCC Subcohorts Stratified by Gender eTable 5. QoL Utility Measured in HCC Patients of NCKUH Stratified by Gender and AJCC 7th Staging (2011-2019) eTable 6. Loss-of-LE and Loss-of-QALE of HCC Patients Stratified by Gender and AJCC 7th Staging, Followed During 2002-2017 eTable 7. Frequency Distribution of HCC Subcohorts Stratified by Gender, Different Underlying Liver Disease, and AJCC 7th Staging eTable 8. Loss-of-LE and Loss-of-QALE of Different HCC Subcohorts Weighted by AJCC 7th Stage Distribution eFigure 10. Adjustment of Age, Sex, and Calendar-Year of Diagnosis by Estimating the Difference-in-Differences of Loss-of-LE eReferences. [file jamanetwopen-e2114680-s001.pdf]

Supplementary Online Content

Kuo SC, Lin CN, Lin YJ, Chen WY, Hwang JS, Wang JD. Optimal intervals of ultrasonography screening for early diagnosis of hepatocellular carcinoma in Taiwan. *JAMA Netw Open*. 2021;4(6):e2114680. doi:10.1001/jamanetworkopen.2021.14680

eFigure 1. Flow Diagram of Study Cohort
eMethods 1. Theoretical Part of the Statistical Methods
eMethods 2. Empirical Part of the Statistical Methods
eFigure 2. Kaplan-Meier Survival Estimates for Stage A Male HCC Patients and the Corresponding Referents
eFigure 3. Logit Transformation of Survival Ratio *W(t)* Between Index Cohort and Referents Fitted with a Restricted Cubic Spline Model
eFigure 4. Rolling-Over Extrapolation of logit[*W(t)*] to Lifetime
eFigure 5. Lifetime Survival Function of Stage A Male HCC Patients and the Age-, Sex-, Calendar Year-Matched Referents
eTable 1. Parameters Selected for Rolling Extrapolation of Survival Functions of HCC Patients
eFigure 6. Kernel-Smoothing Mean of QoL for Stage A Male HCC Patients, From Diagnosis to the Maximum Time *Fq*
eFigure 7. Average QoL of Stage A Male HCC Patients in *K* Months Before Death
eFigure 8. QALE, the Integration of Lifetime Survival Function $\hat{S}(t \mid \text{index})$ and Lifetime QoL Function $\hat{Q}(t \mid \text{index})$
eFigure 9. Loss-of-QALE of the Stage A Male HCC Patients
eTable 2. Parameters Selected for QALE Estimation of HCC Patients
eTable 3. Frequency Distribution of HCC Subcohorts Stratified by Gender, Different Underlying Liver Disease, and BCLC Stage
eTable 4. Baseline Characteristics, Comorbidities, and AJCC 7th Stage Distributions of Different HCC Subcohorts Stratified by Gender
eTable 5. QoL Utility Measured in HCC Patients of NCKUH Stratified by Gender and AJCC 7th Staging (2011-2019)
eTable 6. Loss-of-LE and Loss-of-QALE of HCC Patients Stratified by Gender and AJCC 7th Staging, Followed During 2002-2017
eTable 7. Frequency Distribution of HCC Subcohorts Stratified by Gender, Different Underlying Liver Disease, and AJCC 7th Staging
eTable 8. Loss-of-LE and Loss-of-QALE of Different HCC Subcohorts Weighted by AJCC 7th Stage Distribution
eFigure 10. Adjustment of Age, Sex, and Calendar-Year of Diagnosis by Estimating the Difference-in-Differences of Loss-of-LE
eReferences.

This supplementary material has been provided by the authors to give readers additional information about their work.

© 2021 Kuo SC et al. *JAMA Network Open*.

eFigure 1. Flow Diagram of Study Cohort

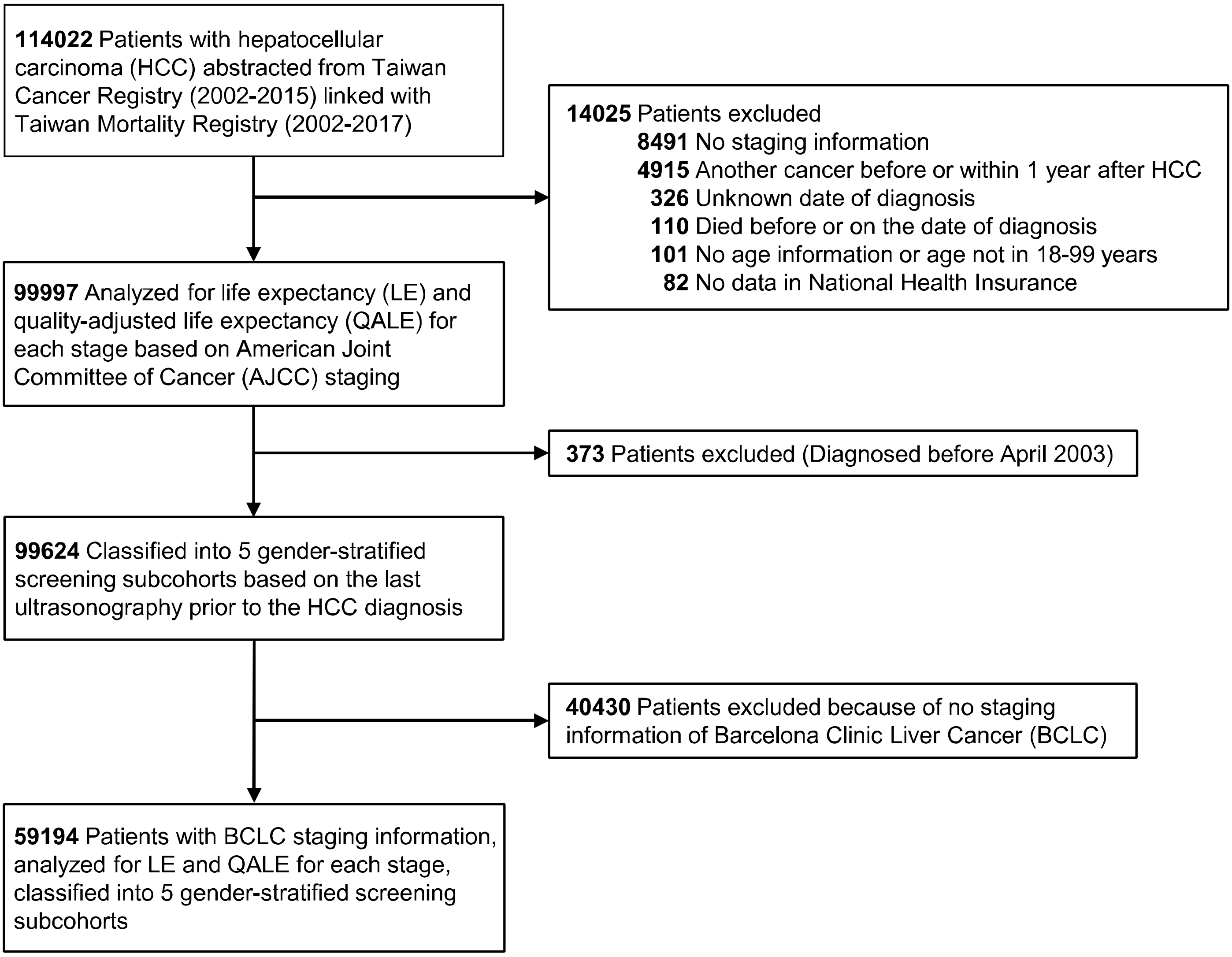

Interlinkages of national databases and exclusion process.

© 2021 Kuo SC et al. *JAMA Network Open*.

eMethods 1. Theoretical Part of the Statistical Methods

Step-by-step Description of Rolling Extrapolation Algorithm

The extrapolation algorithm was carried out by applying the iSQoL2 package which can be freely downloaded using the following link: http://sites.stat.sinica.edu.tw/isQoL/. On the website, one can also download the instruction manual which is a PDF file on how to install the iSQoL2 package and instructions on how to choose parameters and prepare files needed to perform estimations of lifetime survival function, loss-of-LE (life expectancy) and loss-of-QALE (quality-adjusted life expectancy). The details of the rolling extrapolation method were described in Hwang et al[1]. We briefly summarize the main procedures in the following steps:

Step 1: By linkage of the National Health Insurance data with the National Mortality Registry, we could ascertain which cases were still alive, censored or deceased at the end of follow-up (December 2017). The survival datasets of HCC patients stratified by BCLC stage consisted of the year of diagnosis, age, gender, and survival time with censoring indicator for each individual in the index cohort. The survival function of the index cohort, denoted as $S(t|index)$, was estimated using the Kaplan-Meier method for $t \leq F_S$, where F_S is the maximum of follow-up time.

Step 2: For every HCC (hepatocellular carcinoma) patient in each stage, we simulated survival times for age- and sex-matched referents based on the hazard function of the life table of the same calendar year of diagnosis with a fixed ratio to make a dataset size of around 100,000. We then applied the Kaplan-Meier method to the simulated dataset to obtain the survival function of the reference cohort, denoted as $S(t|ref)$.

Step 3: We performed a logit transformation of the relative survival, $W(t) = S(t|index)/S(t|ref)$. The purpose of this transformation was to straighten the relative survival so that it would be easier to conduct extrapolation on $logit[W(t)]$ instead of $W(t)$.

Step 4: We then extrapolated $logit[W(t)]$ with a fitted restricted cubic spline model to the observed period from month 1 to month $F \leq F_S$. Instead of a single extrapolation which extended far beyond F, we extrapolated only one month ahead at a time. We treated the predicted value of the next month as a new "observation" because the $logit[W(t)]$ would approximate to a straight line after a long follow-up. We repeated the extrapolation over and over for the one month ahead of each time by fitting a new restricted cubic spline model to the updated period of same length, dropping the first observation and adding the newly "observed" value to the end. Through such a repeated rolling-over month-by-month extrapolation, we estimated the lifetime survival function of the index cohort by the following equation:

$$\hat{S}(t_{F+k}|index) = S(t_{F+k}|ref) \times \frac{\exp[logit\ W(t_{F+k})]}{1+exp[logit\ W(t_{F+k})]} \ \text{for} \ k = 1, 2, 3, \dots, L - F.$$

Where, F is the last month before starting extrapolation of survival function and L is the month beyond which the survival rate of the HCC patients in each stage is close to zero (say, below 0.01). Typically, we chose a proper F close to F_S, depending on the sparsity of the observed death events.

Estimation of Quality-Adjusted Life Expectancy (QALE) and Loss-of-QALE

The details of the method for estimating QALE and loss-of-QALE were described in Chung et al[2]. We

© 2021 Kuo SC et al. *JAMA Network Open*.

briefly summarize the key procedures in the following. The QALE of the index cohort was estimated from the following equation: $\text{QALE} = \sum_{t=0}^{L} \hat{S}(t|index) \times \hat{Q}(t|index)$, where $\hat{S}(t|index)$ is the lifetime survival function derived from the extrapolation described above and $\hat{Q}(t|index)$ is the QoL (quality of life) function of patients alive at time *t.*

Kernel-Smoothing Method

When QoL measurements from a sample of *n* patients with the index disease are available, the estimate of the mean QoL function of the index cohort, $\hat{Q}(t|\text{index})$, can be obtained using a kernel smoothing method for time *t* from 0 to the maximum assessment time. In notations, $q_i(t)$ represents the QoL utility of the *i*th patient measured in month *t* for $i = 1,2,\ldots,n$. Suppose that patient *i* had been interviewed J_i times at assessment time u_{ij} for $j = 1,\ldots,J_i$ and $u_{ij} \leq F_q$, where F_q is the maximum assessment time. The mean QoL function is estimated by

$$\hat{Q}(t|\text{index}) = \frac{\sum_{i=1}^{n}\sum_{j=1}^{J_i} K\left(\frac{G(u_{ij})-G(t)}{b}\right) \times q_i(u_{ij})}{\sum_{i=1}^{n}\sum_{j=1}^{J_i} K\left(\frac{G(u_{ij})-G(t)}{b}\right)} \quad \text{for } t \leq F_q,$$

where the kernel function $K(u) = 1$, if $|u| \leq 1$, and 0 otherwise. The empirical distribution $G(x)$ is defined as the number of observed assessment times that are less than *x* months divided by the total number, $\sum_{i=1}^{n} J_i$. The bandwidth b is the proportion of data to be used for smoothing, say 5%. The details of the kernel-smoothing method were described in Hwang et al[3].

Extrapolation of QoL Function Beyond Maximum Assessment Time

The right-tailed part of the QoL function produced by the kernel-smoothing method may be biased due to the relatively few observations near the maximum assessment time F_q. Therefore, we started the extrapolation of the mean QoL function at an earlier time point $F_b \leq F_q$, where b is the chosen bandwidth and F_b is the $100 \times (1-b)^{th}$ percentile of all the observed assessment time points. The extrapolation of mean QoL function beyond F_b can be estimated by the following equation: $\hat{Q}(t|index) = \hat{h}(t) \times \hat{Q}_0 + \sum_{k=1}^{K} \prod_{g=0}^{k-1}\left[1-\hat{h}(t+g)\right] \times \hat{h}(t+k) \times \hat{Q}_k + \prod_{g=0}^{k}\left[1-\hat{h}(t+g)\right] \times Q_{K^+}$, where $\hat{h}(t)$ is the estimated hazard of death during month *t* obtained from the extrapolated survival function. The average QoL utility for the subgroup who died in the coming *k* months is denoted by $\hat{Q}_k$ for $k = 0,1,\ldots K$, and $\hat{Q}_{K^+}$ for those who lived beyond the next *K* months. We used the QoL measurements taken at the time close to the maximum assessment time F_q, say, the last *B* months, for estimating $\hat{Q}_k$, which would be the mean QoL function beyond F_b. That is, we first identified the subjects who died between $F_q - B + 1$ and F_q months, and then calculated $\hat{Q}_k$, by averaging utility values of the identified patients who were interviewed k months before death. The $\hat{Q}_{K^+}$ was obtained by taking the average of all QoL measurements of patients who were assessed beyond the $100 \times (1-2b)^{th}$ percentile of all the observed assessment times (F_{2b}), and who also survived at least K months after the assessment.

QoL Function of the Corresponding Referents

In the survival extrapolation procedures, we have already generated a age- and sex-matched reference cohort. To estimate the QoL function of the matched reference cohort, we used the EQ-5D measurements of a representative sample from the 2013 National Health Interview Survey in Taiwan. We transformed these measurements to utility values and calculated the average QoL utility values for each gender and age

group. We used these average QoL utility values to construct a QoL utility function for each individual in the reference cohort from the time 0 to the individual's survival time. The mean QoL function of the reference cohort would be the average of QoL functions of all the surviving individuals. In notation, the mean utility values for each age group and sex, denoted as P (age, sex), was obtained and categorized by each month using the interpolation method. The utility value for the ith generated referent with survival time T_i, age a_i, and sex s_i was calculated by $q_i(t|a_i, s_i) = P(a_i + t, s_i)$ for $t = 0,1, \dots T_i$. We then estimated the QoL function of the reference population by applying the following equation: $\hat{Q}(t|ref) = \frac{1}{\#\{i: T_{i \geq t}\}} \sum_{\{i: T_{i \geq t}\}} q_i(t|a_i, s_i)$ for $t = 0,1, \dots L$, and the QALE of the referents were obtained by $\sum_{t=0}^{L} \hat{S}(t|ref) \times \hat{Q}(t|ref)$.

© 2021 Kuo SC et al. *JAMA Network Open*.

eMethods 2. Empirical Part of the Statistical Methods

Taking male HCC patients in BCLC stage A subgroup as an example, we depicted the following figures to illustrate the extrapolation algorithm. eFigure 2 shows the Kaplan-Meier survival function of the index (stage A, male patients) and the corresponding referents until the end of follow-up. The curve of logit transformation of $W(t)$ and the fitness of a restricted cubic spline model up to the end of follow-up is shown in eFigure 3. eFigure 4 shows the extrapolation of $logit[W(t)]$ to over L= 948 (=12x (100-21)) months, where 21 was the minimum age of stage A male patients. The life-time survival function of both the stage A male HCC patients and the referents were obtained after the extrapolation (eFigure 5). The LE of stage A male patients and the referents is the area under the respective survival curves. The difference between the area of the above two curves is the loss-of-LE of stage A male patients. The specific parameters used in the estimation of LE and loss-of-LE of all the HCC patients are summarized in eTable 2.

We also depicted the following eFigures to illustrate the process of estimating QALE (quality-adjusted life expectancy) and loss-of-QALE, using stage A male HCC patients as an example. eFigure 6 is the kernel-smoothing method for the estimation of QoL function from time 0 (the date of diagnosis) to the maximum assessment time, and the dots scattered on the figure represent every utility measurement obtained for stage A male patients. eFigure 7 presents the average utility values of those who were still alive at K months prior to death. The extrapolation of lifetime QoL function of stage A male patients and the integration of $\hat{S}(t|index)$ and $\hat{Q}(t|index)$ are depicted in eFigure 8. eFigure 9 shows the quality-adjusted survival curve of stage A male patients and that of the corresponding referents. The difference between the area of the above two curves is the loss-of-QALE of stage A male patients. The specific parameters used in the estimation of QALE and loss-of-LE of all the HCC patients are summarized in eTable 3.

© 2021 Kuo SC et al. *JAMA Network Open*.

eFigure 2. Kaplan-Meier Survival Estimates for Stage A Male HCC Patients and the Corresponding Referents

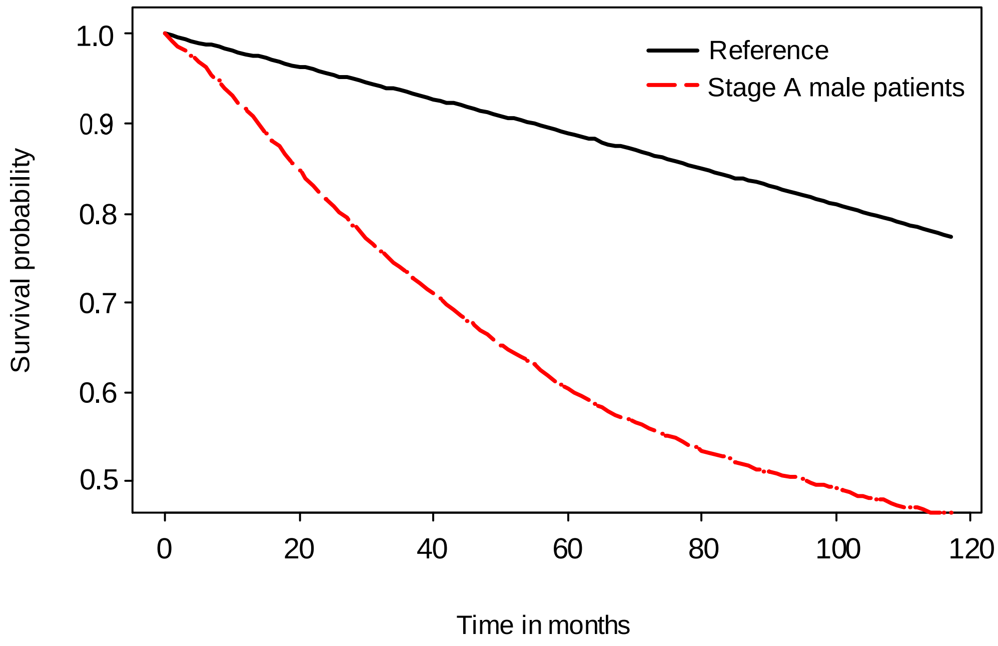

HCC indicates hepatocellular carcinoma. The age-, sex-, and calendar year-matched reference population was simulated from National Vital Statistics, and the survival function of the referents was obtained by applying Kaplan-Meier methods.

eFigure 3. Logit Transformation of Survival Ratio *W(t)* Between Index Cohort and Referents Fitted with a Restricted Cubic Spline Model

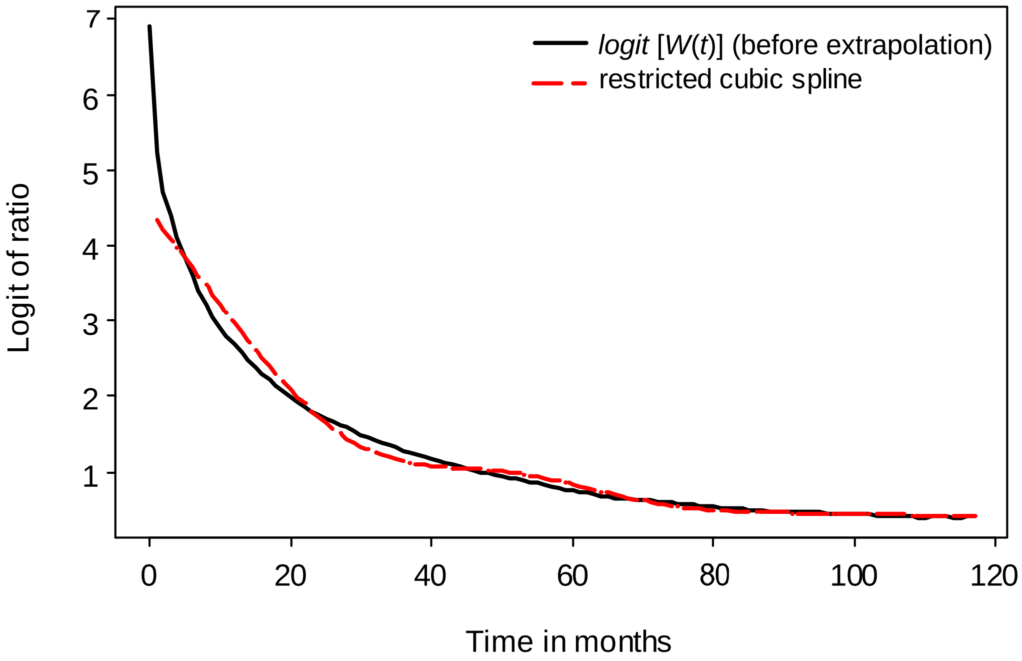

HCC indicates hepatocellular carcinoma. The index cohort indicates stage A male HCC patients. The survival ratio *W(t)* was derived by $\hat{S}(t|index)/\hat{S}(t|ref)$.

© 2021 Kuo SC et al. *JAMA Network Open*.

eFigure 4. Rolling-Over Extrapolation of $logit[W(t)]$ to Lifetime

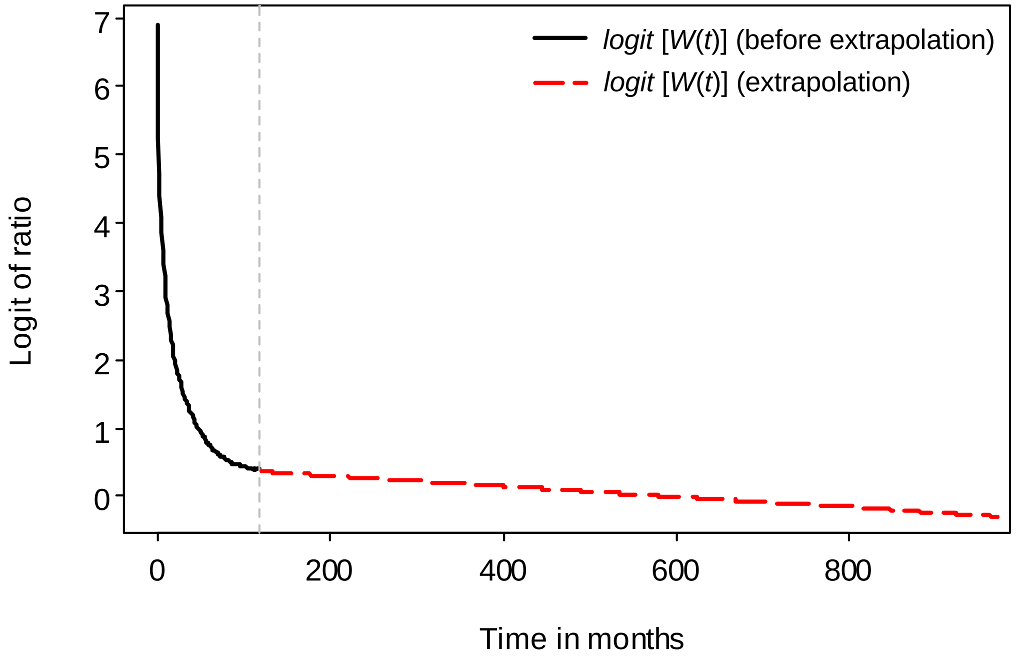

The gray dashed line indicates the last month (*F*) before the extrapolation of $logit[W(t)]$. The extrapolation was conducted over 948 (=12x (100-21)) months, where 21 was the minimum age of the stage A male patients.

© 2021 Kuo SC et al. *JAMA Network Open*.

eFigure 5. Lifetime Survival Functions of Stage A Male HCC Patients and the Age-, Sex-, Calendar Year-Matched Referents

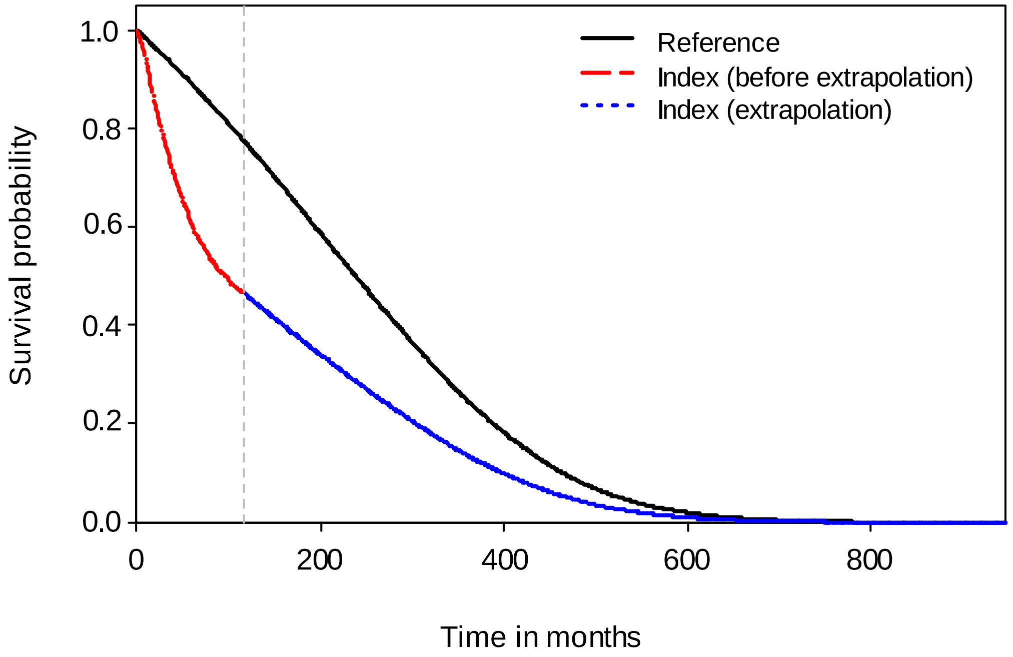

HCC indicates hepatocellular carcinoma. The index cohort indicates stage A male HCC patients. The gray dashed line indicates the last month (*F*) before the extrapolation of survival functions.

© 2021 Kuo SC et al. *JAMA Network Open*.

eTable 1. Parameters Selected for Rolling Extrapolation of Lifetime Survival Functions of HCC Patients

	Male			Female		
**Parameters[a]**	***F*[b]**	***L*[c]**	***H*[d]**	***F***	***L***	***H***
BCLC stage						
0	85	972	24	79	972	24
A	117	948	24	101	948	24
B	105	972	24	95	972	24
C	109	972	24	96	984	24
D	82	984	24	70	972	24

Abbreviation: HCC, hepatocellular carcinoma.

[a] Input parameters required for extrapolating survival function in the R package iSQoL2.

[b] *F* indicates the last month before starting extrapolation of survival function.

[c] *L* is the time for ending extrapolation, which is derived from 12 x (100 – minimum Age in the group).

[d] *H* is the number of time points (months) in the last segment of the restricted cubic splines function for modeling the logit transformation of the survival ratio *W(t)*.

© 2021 Kuo SC et al. *JAMA Network Open*.

eFigure 6. Kernel-Smoothing Mean of QoL for Stage A Male HCC Patients, From Diagnosis to the Maximum Time F_q

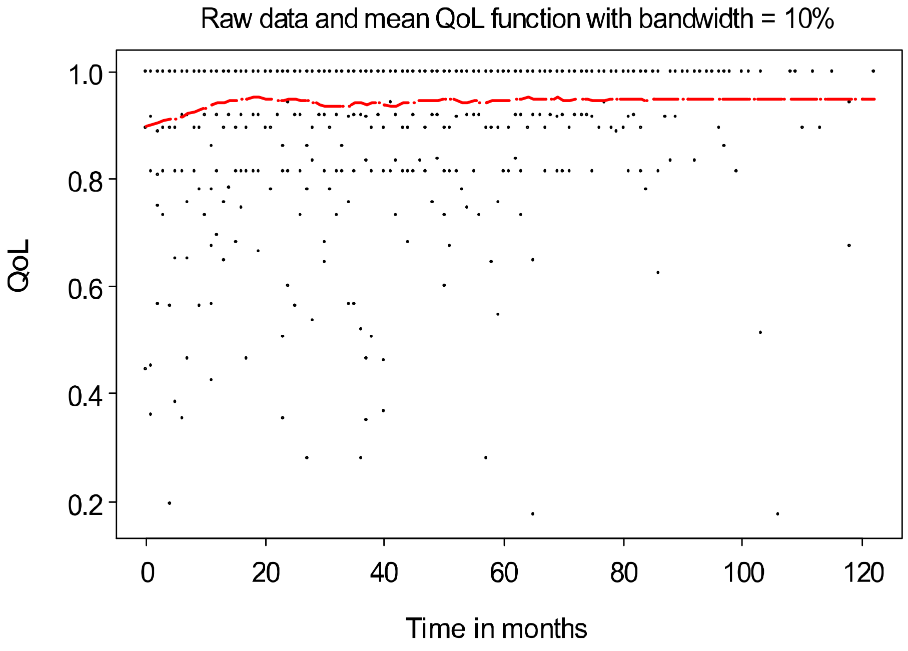

QoL indicates utility values of quality of life; HCC, hepatocellular carcinoma. Every black dot in the figure inidcates a utility measurement taken from stage A male HCC patients, from the date of diagnosis to the maximum assessment time. The red dashed line is the QoL function estimated by kernel-smoothing method.

© 2021 Kuo SC et al. *JAMA Network Open*.

eFigure 7. Average QoL of Stage A Male HCC Patients at *K* Months Before Death

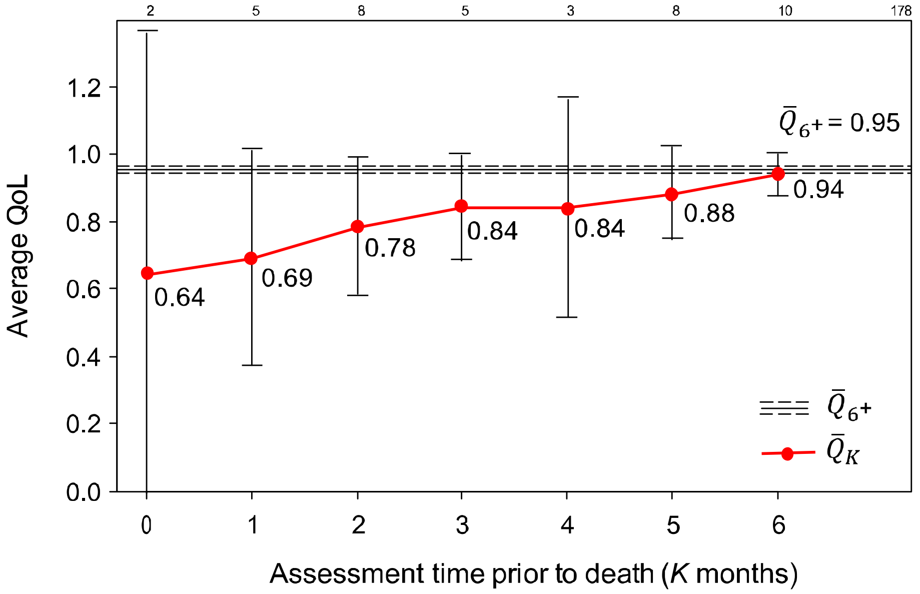

QoL indicates utility values of quality of life; HCC, hepatocellular carcinoma. The error bars at each *K* month indicate the standard error of utility measurement ($\bar{Q}_K$) taken from those who were alive at *K* months prior to death, where *K*=0, 1, … , 6. The horizontal black line and the parallel dashed lines indicate the mean utility measurements ($\bar{Q}_{6^+}$) taken from those who were alive beyond 6 months prior to death (n=178), and the corresponding standard error.

© 2021 Kuo SC et al. *JAMA Network Open*.

eFigure 8. QALE, the Integration of Lifetime Survival Function $\widehat{S}(t|\text{index})$ and Lifetime QoL Function $\widehat{Q}(t|\text{index})$

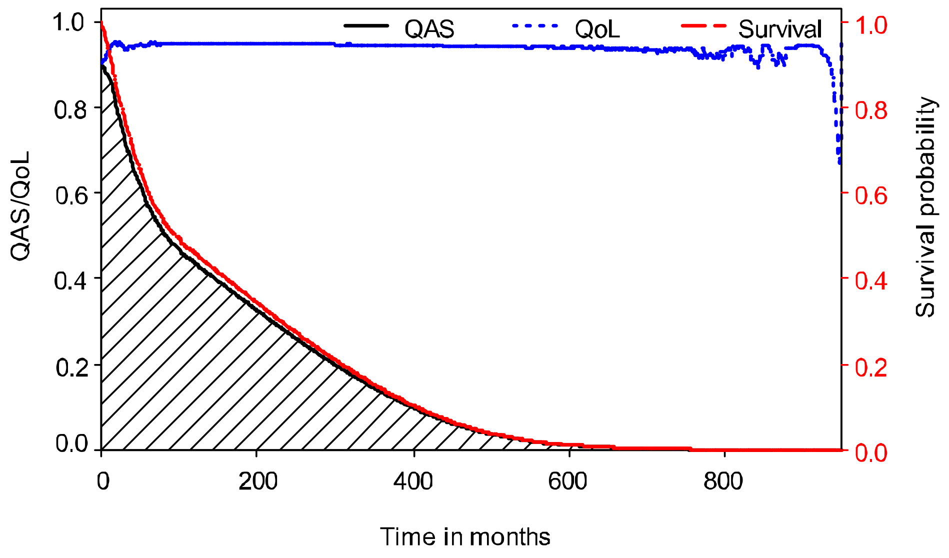

QALE indicates quality-adjusted life expectancy; QoL, utility values of quality of life; QAS, quality-adjusted survival function. The lifetime QoL function (blue dotted line) and survival function (red dashed line) of stage A male HCC patients were obtained after extrapolation, while the QAS was obtained by multiplying QoL utility with survival probability for every time *t*.

eFigure 9. Loss-of-QALE of Stage A Male HCC Patients

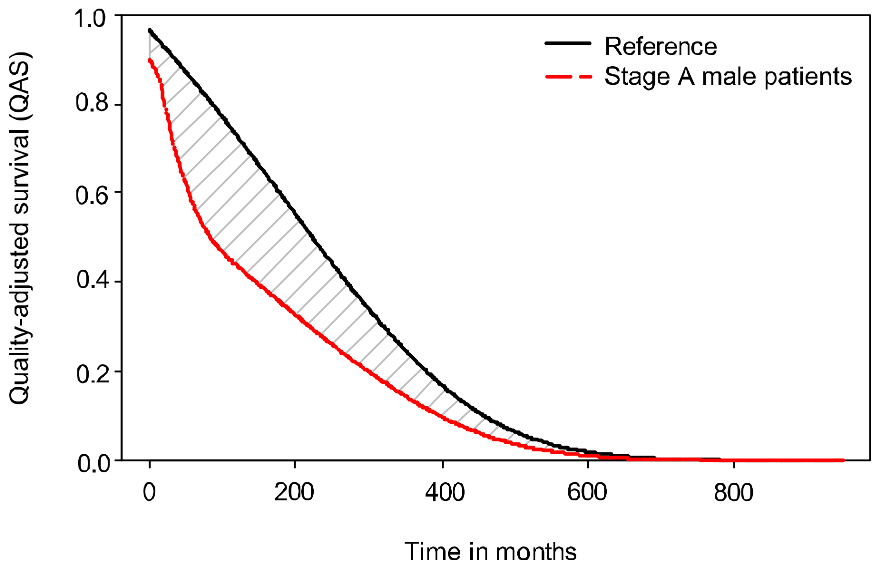

QALE indicates quality-adjusted life expectancy; HCC, hepatocellular carcinoma. The area between the two QAS curves (gray oblique lines) indicates the loss-of-QALE of the stage A male HCC patients compared with the corresponding age-, sex-, and calendar year-matched referents.

© 2021 Kuo SC et al. *JAMA Network Open*.

eTable 2. Parameters Selected for QALE Estimation of HCC Patients

	Male			Female		
**Parameters**[a]	***b***[b]	***K***[c]	***B***[c]	***b***	***K***	***B***
BCLC stage						
0	10	-1	77	10	-1	67
A	10	6	96	10	-1	110
B	10	3	96	10	-1	98
C	10	6	76	15	-1	49
D	15	-1	77	15	-1	49

Abbreviation: QALE, quality-adjusted life expectancy; HCC, hepatocellular carcinoma.

[a] The input parameters required for estimating QALE in the R package iSQoL2.

[b] *b* indicates the bandwidth used in kernel-smoothing method.

[c] *K* and *B* are parameters for the extrapolation of mean QoL function. *K* is set to -1 when the extrapolated mean QoL is assumed constant over time.

© 2021 Kuo SC et al. *JAMA Network Open*.

eTable 3. Frequency Distribution of HCC Subcohorts Stratified by Gender, Different Underlying Liver Disease, and BCLC Stage

	Male No. (%)					Female No. (%)				
**Subcohorts[a]**	**6M**	**12M**	**24M**	**36M**	**>36M**	**6M**	**12M**	**24M**	**36M**	**>36M**
Hepatitis B										
0	865 (12.10)	132 (7.95)	59 (3.97)	33 (3.41)	164 (1.65)	309 (14.42)	43 (9.45)	19 (5.12)	11 (5.26)	38 (2.20)
A	3881 (54.30)	740 (44.58)	552 (37.17)	278 (28.75)	1567 (15.73)	1246 (58.14)	222 (48.79)	152 (40.97)	69 (33.01)	342 (19.79)
B	1188 (16.62)	398 (23.98)	417 (28.08)	281 (29.06)	2871 (28.82)	302 (14.09)	86 (18.90)	98 (26.42)	62 (29.67)	496 (28.70)
C	948 (13.26)	306 (18.43)	358 (24.11)	297 (30.71)	4370 (43.86)	188 (8.77)	79 (17.36)	80 (21.56)	50 (23.92)	654 (37.85)
D	265 (3.71)	84 (5.06)	99 (6.67)	78 (8.07)	991 (9.95)	98 (4.57)	25 (5.49)	22 (5.93)	17 (8.13)	198 (11.46)
Hepatitis C with cirrhosis										
0	429 (11.78)	53 (5.94)	17 (2.44)	11 (2.44)	49 (1.62)	493 (12.93)	60 (8.08)	24 (4.44)	11 (3.68)	42 (2.85)
A	1912 (52.48)	388 (43.45)	280 (40.23)	117 (25.94)	575 (18.97)	2125 (55.73)	358 (48.18)	224 (41.48)	95 (31.77)	374 (25.34)
B	636 (17.46)	207 (23.18)	179 (25.72)	135 (29.93)	853 (28.14)	588 (15.42)	130 (17.50)	139 (25.74)	76 (25.42)	366 (24.80)
C	480 (13.18)	192 (21.50)	175 (25.14)	148 (32.82)	1225 (40.42)	444 (11.64)	151 (20.32)	106 (19.63)	85 (28.43)	494 (33.47)
D	186 (5.11)	53 (5.94)	45 (6.47)	40 (8.87)	329 (10.85)	163 (4.27)	44 (5.92)	47 (8.70)	32 (10.70)	200 (13.55)
Hepatitis C no cirrhosis										
0	158 (14.02)	31 (9.12)	11 (3.46)	8 (4.17)	47 (3.97)	133 (16.84)	26 (11.61)	16 (10.81)	9 (7.89)	25 (4.78)
A	669 (59.36)	186 (54.71)	159 (50.00)	66 (34.38)	294 (24.85)	486 (61.52)	124 (55.36)	66 (44.59)	59 (51.75)	148 (28.30)
B	177 (15.71)	78 (22.94)	89 (27.99)	63 (32.81)	398 (33.64)	92 (11.65)	34 (15.18)	36 (24.32)	20 (17.54)	170 (32.50)
C	113 (10.03)	40 (11.76)	49 (15.41)	46 (23.96)	362 (30.60)	68 (8.61)	33 (14.73)	25 (16.89)	20 (17.54)	135 (25.81)

© 2021 Kuo SC et al. *JAMA Network Open*.

D	10 (0.89)	5 (1.47)	10 (3.14)	9 (4.69)	82 (6.93)	11 (1.39)	7 (3.13)	5 (3.38)	6 (5.26)	45 (8.60)
Cirrhosis										
0	1078 (10.92)	131 (5.91)	60 (2.95)	32 (2.43)	173 (1.40)	727 (12.51)	87 (7.53)	35 (3.79)	19 (3.49)	72 (2.12)
A	4971 (50.34)	886 (39.96)	683 (33.56)	319 (24.22)	1761 (14.23)	3190 (54.90)	519 (44.94)	351 (38.03)	155 (28.49)	682 (20.04)
B	1662 (16.83)	523 (23.59)	519 (25.50)	354 (26.88)	2965 (23.97)	862 (14.83)	207 (17.92)	220 (23.84)	132 (24.26)	787 (23.13)
C	1534 (15.53)	494 (22.28)	554 (27.22)	458 (34.78)	5632 (45.52)	698 (12.01)	244 (21.13)	212 (22.97)	164 (30.15)	1265 (37.17)
D	630 (6.38)	183 (8.25)	219 (10.76)	154 (11.69)	1841 (14.88)	334 (5.75)	98 (8.48)	105 (11.38)	74 (13.60)	597 (17.54)
No underlying liver disease										
0	30 (4.48)	7 (2.43)	7 (1.76)	5 (1.51)	37 (0.99)	28 (9.96)	11 (6.83)	8 (4.42)	4 (3.17)	17 (1.12)
A	278 (41.55)	94 (32.64)	110 (27.64)	73 (22.05)	485 (13.02)	108 (38.43)	57 (35.40)	49 (27.07)	29 (23.02)	235 (15.44)
B	153 (22.87)	71 (24.65)	96 (24.12)	112 (33.84)	1096 (29.42)	41 (14.59)	33 (20.50)	46 (25.41)	33 (26.19)	385 (25.30)
C	125 (18.68)	70 (24.31)	113 (28.39)	79 (23.87)	1380 (37.05)	67 (23.84)	34 (21.12)	47 (25.97)	36 (28.57)	561 (36.86)
D	83 (12.41)	46 (15.97)	72 (18.09)	62 (18.73)	727 (19.52)	37 (13.17)	26 (16.15)	31 (17.13)	24 (19.05)	324 (21.29)

Abbreviations: HCC, hepatocellular carcinoma; BCLC, Barcelona Clinic Liver Cancer.

[a] 5 different gender-stratified subcohorts were categorized based on the timing of the last ultrasonography before the index date (90 days prior to the HCC diagnosis). Subcohorts: 6M, 0-6 months; 12M, 6-12 months; 24M, 12-24 months; 36M, 24-36 months; >36M, no screening within 3 years prior to the index date.

© 2021 Kuo SC et al. *JAMA Network Open*.

eTable 4. Baseline Characteristics, Comorbidities, and AJCC 7th Stage Distributions of Different HCC Subcohorts Stratified by Gender

	Male					Female				
**Subcohorts[a]**	**6M**	**12M**	**24M**	**36M**	**>36M**	**6M**	**12M**	**24M**	**36M**	**>36M**
Number of cases	21434	5846	5765	3794	34919	11488	2778	2350	1473	9777
Age (mean±SD)	61.8±11.6	62.5±12.2	63.1±12.5	63.7±12.8	61.0±13.6	67.6±9.7	68.4±10.4	69.0±11.0	69.3±11.3	68.4±13.6
No. of ultrasonography[b]	6.1±3.8	3.4±2.3	2.1±1.4	1.3±0.7	0.0±0.0	6.7±3.9	3.8±2.4	2.3±1.5	1.3±0.7	0.0±0.0
Underlying liver disease (%)										
Hepatitis B	11231 (52.40)	2873 (49.14)	2691 (46.68)	1709 (45.04)	17084 (48.92)	3352 (29.18)	767 (27.61)	633 (26.94)	372 (25.25)	2824 (28.88)
Hepatitis C	7746 (36.14)	2048 (35.03)	1783 (30.93)	1148 (30.26)	7036 (20.15)	7108 (61.87)	1600 (57.60)	1197 (50.94)	716 (48.61)	3166 (32.38)
Liver cirrhosis	16177 (75.47)	3934 (67.29)	3758 (65.19)	2419 (63.76)	21505 (61.59)	9327 (81.19)	1966 (70.77)	1626 (69.19)	956 (64.90)	5554 (56.81)
Alcoholic liver disease	2718 (12.68)	670 (11.46)	667 (11.57)	401 (10.57)	3542 (10.14)	270 (2.35)	54 (1.94)	49 (2.09)	26 (1.77)	141 (1.44)
Other comorbidities (%)										
Ischemic heart disease	1855 (8.65)	520 (8.89)	525 (9.11)	359 (9.46)	2221 (6.36)	1086 (9.45)	268 (9.65)	225 (9.57)	155 (10.52)	750 (7.67)
Heart failure	542 (2.53)	179 (3.06)	181 (3.14)	125 (3.29)	965 (2.76)	441 (3.84)	118 (4.25)	121 (5.15)	78 (5.30)	482 (4.93)
Diabetes mellitus	6858 (32.00)	1785 (30.53)	1762 (30.56)	1105 (29.12)	7680 (21.99)	3867 (33.66)	933 (33.59)	795 (33.83)	520 (35.30)	2878 (29.44)
Cerebrovascular disease	1314 (6.13)	383 (6.55)	407 (7.06)	272 (7.17)	1999 (5.72)	668 (5.81)	198 (7.13)	162 (6.89)	111 (7.54)	613 (6.27)
Chronic kidney disease	1489 (6.95)	476 (8.14)	369 (6.40)	294 (7.75)	1507 (4.32)	738 (6.42)	195 (7.02)	188 (8.00)	135 (9.16)	523 (5.35)
COPD	892 (4.16)	277 (4.74)	284 (4.93)	192 (5.06)	1140 (3.26)	332 (2.89)	90 (3.24)	80 (3.40)	42 (2.85)	262 (2.68)
AJCC stage distribution (%)										

© 2021 Kuo SC et al. *JAMA Network Open*.

I	9956 (46.45)	2264 (38.73)	1839 (31.90)	1028 (27.10)	6364 (18.23)	6043 (52.60)	1233 (44.38)	911 (38.77)	490 (33.27)	2368 (24.22)
II	7087 (33.06)	1743 (29.82)	1532 (26.57)	839 (22.11)	5655 (16.19)	3797 (33.05)	856 (30.81)	633 (26.94)	347 (23.56)	1782 (18.23)
III	3271 (15.26)	1327 (22.70)	1666 (28.90)	1341 (35.35)	15016 (43.00)	1200 (10.45)	484 (17.42)	573 (24.38)	430 (29.19)	3685 (37.69)
IV	1120 (5.23)	512 (8.76)	728 (12.63)	586 (15.45)	7884 (22.58)	448 (3.90)	205 (7.38)	233 (9.91)	206 (13.99)	1942 (19.86)

Abbreviations: AJCC, American Joint Committee on Cancer; HCC, hepatocellular carcinoma; COPD, chronic obstructive pulmonary disease;

[a] 5 different gender-stratified subcohorts were categorized based on the timing of the last ultrasonography before the index date (90 days prior to the HCC diagnosis). Subcohorts: 6M, 0-6 months; 12M, 6-12 months; 24M, 12-24 months; 36M, 24-36 months; >36M, no screening within 3 years prior to the index date.

[b] Abdominal ultrasonography numbers within 3 years prior to the index date are presented with mean and standard deviation.

© 2021 Kuo SC et al. *JAMA Network Open*.

eTable 5. QoL Utility Measured in HCC Patients from NCKUH Stratified by Gender and AJCC 7th Staging (2011-2019)

Stage	Gender	No. of cases	Age at diagnosis (mean±SD)	Measurements in the 1st year		Measurements in the 2nd year		Measurements in the 3rd year+	
				No.	Mean utility[a]	No.	Mean utility	No.	Mean utility [c]
I	Male	370	58.3±10.4	286	0.92±0.14	285	0.95±0.10	1141	0.93±0.12
	Female	149	62.7±12.0	116	0.84±0.18	94	0.89±0.16	501	0.88±0.17
II	Male	338	57.8±10.3	307	0.91±0.14	313	0.94±0.12	1021	0.93±0.12
	Female	109	64.1±9.5	82	0.87±0.17	85	0.91±0.14	268	0.89±0.15
III	Male	189	58.5±10.9	232	0.87±0.19	134	0.90±0.17	244	0.91±0.14
	Female	40	59.0±11.1	52	0.87±0.14	30	0.86±0.11	82	0.91±0.15
IV	Male	35	53.9±11.8	49	0.75±0.25	15	0.79±0.25	17	0.87±0.16
	Female	6	56.5±11.6	6	0.70±0.27[b]	0		0	

Abbreviations: HCC, hepatocellular carcinoma; QoL, quality of life; NCKUH, National Cheng Kung University Hospital; AJCC, American Joint Committee on Cancer.

[a] Utility values were repeatedly measured using European quality of life-5 dimensions (EQ-5D) questionnaires and converted into Taiwan value set[25].

[b] There were only 6 EQ-5D measurements of stage IV women and all values were measured within the first year after diagnosis.

[c] 3rd year+ mean utility denotes the average value of all the utilities measured from the beginning of the third year till the end of follow-up.

© 2021 Kuo SC et al. *JAMA Network Open*.

eTable 6. Loss-of-LE and Loss-of-QALE of HCC Patients Stratified by Gender and AJCC 7th Staging, Followed During 2002-2017

Stage	Gender	No. of cases	Age at diagnosis (mean±SD)	Censored cases (%)	LE (95% CI) (years)	Loss-of-LE (95% CI) (years)	QALE (95% CI) (QALYs)	Loss-of-QALE (95% CI) (QALYs)
I	Male	21517	62.6±12.4	9683 (45.0)	10.2 (9.3-11.1)	10.4 (9.4-11.3)	9.5 (8.6-10.3)	10.0 (9.2-10.8)
	Female	11077	67.9±10.9	4553 (41.1)	8.2 (7.5-8.9)	11.2 (10.5-11.9)	7.2 (6.5-7.9)	9.8 (9.1-10.5)
II	Male	16963	61.8±12.1	5428 (32.0)	7.7 (7.0-8.4)	13.4 (12.7-14.1)	7.2 (6.5-7.9)	12.8 (12.1-13.5)
	Female	7460	67.6±10.5	2074 (27.8)	5.8 (5.2-6.3)	13.8 (13.2-14.4)	5.1 (4.6-5.6)	12.0 (11.4-12.6)
III	Male	22699	61.4±13.4	1816 (8.0)	2.6 (2.3-2.9)	18.9 (18.6-19.3)	2.4 (2.0-2.7)	18.1 (17.8-18.4)
	Female	6409	69.1±12.8	455 (7.1)	2.2 (1.7-2.7)	16.5 (15.9-17.1)	1.9 (1.5-2.4)	14.4 (13.8-14.9)
IV	Male	10836	60.3±13.4	238 (2.2)	0.8 (0.7-0.9)	21.6 (21.4-21.8)	0.6 (0.5-0.8)	20.6 (20.4-20.9)
	Female	3036	68.7±12.9	61 (2.0)	0.7 (0.6-0.8)	18.3 (17.9-18.7)	0.5 [a]	16.1 [a]

Abbreviations: LE, life expectancy; QALE, quality-adjusted life expectancy; HCC, hepatocellular carcinoma; AJCC, American Joint Committee on Cancer; QALYs, quality-adjusted life years.

[a] The number of EQ-5D measurements of stage IV women were too few (total 6 measurements), so we took the average of all measurements and assumed a constant utility value throughout lifetime. Standard error of the mean could not be calculated in this situation.

© 2021 Kuo SC et al. *JAMA Network Open*.

eTable 7. Frequency Distribution of HCC Subcohorts Stratified by Gender, Different Underlying Liver Disease, and AJCC 7th Staging

	Male No. (%)					Female No.(%)				
**Subcohorts[a]**	**6M**	**12M**	**24M**	**36M**	**>36M**	**6M**	**12M**	**24M**	**36M**	**>36M**
Hepatitis B										
I	5378 (47.89)	1142 (39.75)	870 (32.33)	462 (27.03)	2950 (17.27)	1826 (54.47)	363 (47.33)	262 (41.39)	137 (36.83)	667 (23.62)
II	3732 (33.23)	863 (30.04)	721 (26.79)	405 (23.70)	2850 (16.68)	1127 (33.62)	242 (31.55)	181 (28.59)	99 (26.61)	554 (19.62)
III	1615 (14.38)	647 (22.52)	804 (29.88)	605 (35.40)	7650 (44.78)	297 (8.86)	122 (15.91)	143 (22.59)	96 (25.81)	1117 (39.55)
IV	506 (4.51)	221 (7.69)	296 (11.00)	237 (13.87)	3634 (21.27)	102 (3.04)	40 (5.22)	47 (7.42)	40 (10.75)	486 (17.21)
Hepatitis C										
I	3699 (47.75)	819 (39.99)	610 (34.21)	321 (27.96)	1477 (20.99)	3836 (53.97)	712 (44.50)	494 (41.27)	257 (35.89)	874 (27.61)
II	2771 (35.77)	686 (33.50)	562 (31.52)	278 (24.22)	1513 (21.50)	2439 (34.31)	550 (34.38)	356 (29.74)	191 (26.68)	761 (24.04)
III	959 (12.38)	418 (20.41)	434 (24.34)	393 (34.23)	2843 (40.41)	615 (8.65)	253 (15.81)	259 (21.64)	190 (26.54)	1089 (34.40)
IV	317 (4.09)	125 (6.10)	177 (9.93)	156 (13.59)	1203 (17.10)	218 (3.07)	85 (5.31)	88 (7.35)	78 (10.89)	442 (13.96)
Cirrhosis										
I	7195 (44.48)	1386 (35.23)	1087 (28.92)	562 (23.23)	3222 (14.98)	4809 (51.56)	848 (43.13)	595 (36.59)	296 (30.96)	1230 (22.15)
II	5520 (34.12)	1199 (30.48)	1022 (27.20)	551 (22.78)	3488 (16.22)	3189 (34.19)	623 (31.69)	463 (28.47)	222 (23.22)	1116 (20.09)
III	2644 (16.34)	1003 (25.50)	1165 (31.00)	927 (38.32)	9937 (46.21)	994 (10.66)	362 (18.41)	422 (25.95)	304 (31.80)	2193 (39.49)
IV	818 (5.06)	346 (8.80)	484 (12.88)	379 (15.67)	4858 (22.59)	335 (3.59)	133 (6.77)	146 (8.98)	134 (14.02)	1015 (18.28)
No underlying liver disease										
I	530 (41.70)	208 (36.75)	252 (32.68)	182 (31.01)	1499 (22.36)	244 (44.85)	118 (44.36)	122 (38.61)	65 (30.23)	616 (24.43)

© 2021 Kuo SC et al. *JAMA Network Open*.

II	307 (24.15)	139 (24.56)	139 (18.03)	93 (15.84)	859 (12.82)	126 (23.16)	53 (19.92)	51 (16.14)	34 (15.81)	328 (13.01)
III	275 (21.64)	130 (22.97)	238 (30.87)	203 (34.58)	2547 (38.00)	105 (19.30)	47 (17.67)	92 (29.11)	68 (31.63)	904 (35.84)
IV	159 (12.51)	89 (15.72)	142 (18.42)	109 (18.57)	1798 (26.82)	69 (12.68)	48 (18.05)	51 (16.14)	48 (22.33)	674 (26.72)

Abbreviations: HCC, hepatocellular carcinoma; AJCC, American Joint Committee on Cancer.

[a] 5 different gender-stratified subcohorts were categorized based on the timing of the last ultrasonography before the index date (90 days prior to the HCC diagnosis). Subcohorts: 6M, 0-6 months; 12M, 6-12 months; 24M, 12-24 months; 36M, 24-36 months; >36M, no screening within 3 years prior to the index date.

[b] Patients without any of the underlying liver disease including hepatitis B, hepatitis C, liver cirrhosis, and alcoholic liver disease were included in the "No underlying liver disease" subgroup.

© 2021 Kuo SC et al. *JAMA Network Open*.

eTable 8. Loss-of-LE and Loss-of-QALE of Different HCC Subcohorts Weighted by AJCC 7th Stage Distribution

Subcohorts[a]		6M	12M	24M	36M	>36M
All subjects						
Male	Loss-of-LE	13.3 (12.7-13.8)	14.2 (13.7-14.7)	15.1 (14.7-15.5)	15.8 (15.5-16.1)	17.1 (16.8-17.4)
	Loss- of-QALE	12.7 (12.2-13.2)	13.6 (13.2-14.0)	14.4 (14.0-14.8)	15.1 (14.8-15.5)	16.3 (16.1-16.6)
Female	Loss-of-LE	12.9 (12.4-13.3)	13.4 (13.1-13.8)	13.9 (13.5-14.3)	14.4 (14.0-14.7)	15.1 (14.8-15.4)
	Loss- of-QALE	11.2 (10.8-11.7)	11.7 (11.4-12.1)	12.1 (11.8-12.5)	12.5 (12.2-12.8)	13.2 (12.9-13.5)
Hepatitis B						
Male	Loss-of-LE	13.1 (12.6-13.7)	14.1 (13.6-14.5)	15.0 (14.6-15.4)	15.7 (15.3-16.0)	17.1 (16.8-17.4)
	Loss- of-QALE	12.6 (12.1-13.1)	13.5 (13.0-13.9)	14.3 (14.0-14.7)	15.0 (14.7-15.3)	16.3 (16.1-16.6)
Female	Loss-of-LE	12.7 (12.3-13.2)	13.2 (12.8-13.6)	13.7 (13.3-14.0)	14.0 (13.7-14.4)	15.0 (14.7-15.4)
	Loss- of-QALE	11.1 (10.7-11.5)	11.5 (11.2-11.9)	11.9 (11.6-12.3)	12.2 (11.9-12.6)	13.1 (12.8-13.4)
Hepatitis C						
Male	Loss-of-LE	13.0 (12.4-13.5)	13.8 (13.3-14.3)	14.5 (14.1-15.0)	15.6 (15.2-15.9)	16.4 (16.1-16.7)
	Loss- of-QALE	12.4 (11.9-13.0)	13.2 (12.8-13.7)	13.9 (13.5-14.3)	14.9 (14.5-15.2)	15.7 (15.4-16.0)
Female	Loss-of-LE	12.8 (12.3-13.2)	13.3 (12.9-13.7)	13.6 (13.3-14.0)	14.1 (13.7-14.4)	14.6 (14.3-15.0)
	Loss- of-QALE	11.1 (10.7-11.6)	11.6 (11.2-12.0)	11.9 (11.5-12.3)	12.3 (11.9-12.6)	12.8 (12.5-13.1)
All patients with cirrhosis						
Male	Loss-of-LE	13.4 (12.9-13.9)	14.5 (14.0-14.9)	15.3 (14.9-15.7)	16.1 (15.8-16.4)	17.4 (17.1-17.6)
	Loss- of-QALE	12.8 (12.3-13.3)	13.9 (13.4-14.3)	14.6 (14.3-15.0)	15.4 (15.1-15.7)	16.6 (16.3-16.8)
Female	Loss-of-LE	12.9 (12.5-13.3)	13.5 (13.1-13.9)	14.0 (13.6-14.3)	14.5 (14.1-14.8)	15.1 (14.8-15.4)

© 2021 Kuo SC et al. *JAMA Network Open*.

	Loss- of-QALE	11.3 (10.8-11.7)	11.8 (11.4-12.1)	12.2 (11.8-12.5)	12.6 (12.3-13.0)	13.2 (12.9-13.5)
No underlying disease						
Male	Loss-of-LE	14.4 (13.9-14.8)	14.8 (14.4-15.3)	15.6 (15.3-16.0)	15.9 (15.5-16.2)	17.0 (16.7-17.3)
	Loss- of-QALE	13.8 (13.3-14.2)	14.2 (13.8-14.6)	15.0 (14.6-15.3)	15.2 (14.9-15.5)	16.3 (16.0-16.5)
Female	Loss-of-LE	13.7 (13.3-14.1)	13.9 (13.6-14.3)	14.3 (14.0-14.6)	14.9 (14.6-15.2)	15.3 (15.0-15.6)
	Loss- of-QALE	12.0 (11.6-12.3)	12.2 (11.8-12.5)	12.5 (12.2-12.8)	13.0 (12.7-13.3)	13.4 (13.1-13.7)

Abbreviations: LE, life expectancy; QALE, quality-adjusted life expectancy; HCC, hepatocellular carcinoma; AJCC, American Joint Committee on Cancer.

[a] 5 different gender-stratified subcohorts were categorized based on the timing of the last ultrasonography before the index date (90 days prior to the HCC diagnosis). Subcohorts: 6M, 0-6 months; 12M, 6-12 months; 24M, 12-24 months; 36M, 24-36 months; >36M, no screening within 3 years prior to the index date.

[b] Patients without any of the underlying liver disease including hepatitis B, hepatitis C, liver cirrhosis, and alcoholic liver disease were included in the “No underlying liver disease” subgroup.

© 2021 Kuo SC et al. *JAMA Network Open*.

eFigure 10. Adjustment of Age, Sex, and Calendar-year of Diagnosis by Estimating the Difference-in-Differences of Loss-of-LE

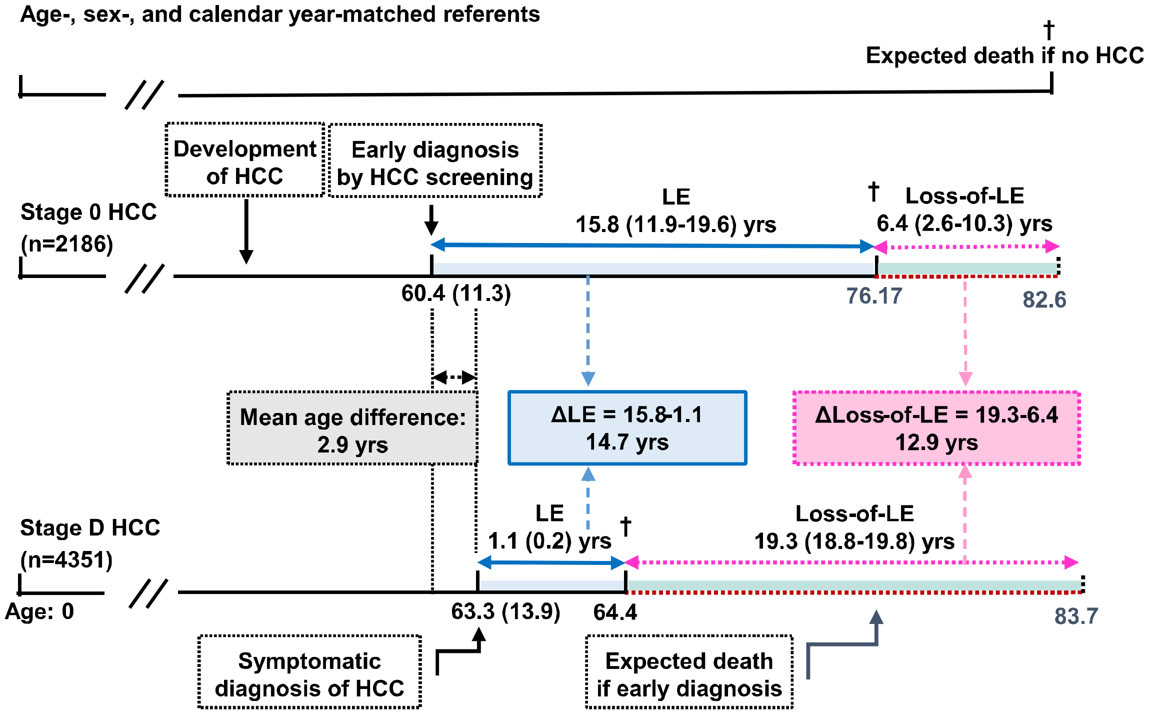

LE indicates life expectancy; HCC, hepatocellular carcinoma; yrs, years. Typically, screening detected patients had higher chances of being diagnosed at an earlier stage and were younger than those diagnosed symptomatically. Assuming that all stage D male HCC patients were diagnosed symptomatically at a mean age of 63.3 and all stage 0 male patients were detected by screening at a mean age of 60.4, the average gain in life expectancy (LE) would be 15.8-1.1=14.7 years. However, if we took different age, sex, year of diagnosis, and comorbidities into consideration and compared the loss-of-LE, the average savings of loss-of-LE would be 19.3-6.4=12.9 years, which implies an adjustment for lead-time bias, or, different age distributions for earlier diagnosis. The values in parentheses of age and LE/loss-of-LE denote the standard deviations and 95% confidence intervals, respectively.

© 2021 Kuo SC et al. *JAMA Network Open*.

eReferences.

1. Hwang JS, Hu TH, Lee LJ, Wang JD. Estimating lifetime medical costs from censored claims data. *Health Econ.* 2017;26(12):e332-e344. [cited as No.22 in the text]
2. Chung CH, Hu TH, Wang JD, Hwang JS. Estimation of Quality-Adjusted Life Expectancy of Patients With Oral Cancer: Integration of Lifetime Survival With Repeated Quality-of-Life Measurements. *Value Health Reg Issues.* 2020;21:59-65. [cited as No.29 in the text]
3. Hwang JS, Tsauo JY, Wang JD. Estimation of expected quality adjusted survival by cross-sectional survey. *Stat Med.* 1996;15(1):93-102. [cited as No.26 in the text]

© 2021 Kuo SC et al. *JAMA Network Open*.
